# “Subpial Fan Cell” — A Class of Calretinin Neuron in Layer 1 of Adult Monkey Prefrontal Cortex

**DOI:** 10.3389/fnana.2016.00028

**Published:** 2016-04-13

**Authors:** Paul L. A. Gabbott

**Affiliations:** ^1^Neural Architectonics CentreOxford, UK; ^2^Department of Life, Health, and Chemical Sciences, The Open UniversityMilton Keynes, UK; ^3^University Department of Pharmacology, University of OxfordOxford, UK

**Keywords:** excitation, connectivity, apical dendritic tufts, integration, minicolumn, development

## Abstract

Layer 1 of the cortex contains populations of neurochemically distinct neurons and afferent fibers which markedly affect neural activity in the apical dendritic tufts of pyramidal cells. Understanding the causal mechanisms requires knowledge of the cellular architecture and synaptic organization of layer 1. This study has identified eight morphological classes of calretinin immunopositive (CRet+) neurons (including Cajal-Retzius cells) in layer 1 of the prefrontal cortex (PFC) in adult monkey *(Macaca fasicularis)*, with a distinct class — termed “*subpial fan (SPF) cell”* — described in detail. SPF cells were rare horizontal unipolar CRet+ cells located directly beneath the pia with a single thick primary dendrite that branched into a characteristic fan-like dendritic tree tangential to the pial surface. Dendrites had spines, filamentous processes and thorny branchlets. SPF cells lay millimeters apart with intralaminar axons that ramified widely in upper layer 1. Such cells were GABA immunonegative (-) and occurred in areas beyond PFC. Interspersed amidst SPF cells displaying normal structural integrity were degenerating CRet+ neurons (including SPF cells) and clumps of lipofuscin-rich cellular debris. The number of degenerating SPF cells increased during adulthood. Ultrastructural analyses indicated SPF cell somata received asymmetric (A — presumed excitatory) and symmetric (S — presumed inhibitory) synaptic contacts. Proximal dendritic shafts received mainly S-type and distal shafts mostly A-type input. All dendritic thorns and most dendritic spines received both synapse types. The tangential areal density of SPF cell axonal varicosities varied radially from parent somata — with dense clusters in more distal zones. All boutons formed A-type contacts with CRet- structures. The main post-synaptic targets were dendritic shafts (67%; mostly spine-bearing) and dendritic spines (24%). SPF-SPF cell innervation was not observed. Morphometry of SPF cells indicated a unique class of CRet+/GABA- neuron in adult monkey PFC — possibly a subtype of persisting Cajal-Retzius cell. The distribution and connectivity of SPF cells suggest they act as integrative hubs in upper layer 1 during postnatal maturation. The main synaptic output of SPF cells likely provides a transminicolumnar excitatory influence across swathes of apical dendritic tufts — thus affecting information processing in discrete patches of layer 1 in adult monkey PFC.

## Introduction

The early Golgi-impregnation studies of Ramón y Cajal (1890, 1891, 1899a,b,c), Retzius (1893, 1894), and others (Kölliker, 1894; Campbell, 1905; Ranke, 1910; Oppermann, 1929) identified the wide morphological variety of neurons in layer 1 (molecular/plexiform layer) present during the pre- and postnatal development of the cerebral cortex in several mammalian species, including humans — with the most studied family of neurons being the horizontal cells described by Ramón y Cajal and by Retzius (Fairén et al., 2002; Gil et al., 2014; Martinez-Cerdeno and Noctor, 2014; Marín-Padilla, 2015). Subsequent studies have refined and extended morphological descriptions of the “special” *Cajal'sche Zellen* (Retzius, 1894) and the *Retzius'sche Zellen* (Kölliker, 1894,^1^) and other neuron phenotypes in layer 1 (for example: Marín-Padilla, 1984, 1998, 2015; Huntley and Jones, 1990; Frotscher, 1998; Meyer et al., 1999; Fairén et al., 2002; Rakic and Zečević, 2003; Soriano and Del Río, 2005; Kirischuk et al., 2014; Martinez-Cerdeno and Noctor, 2014; Lee et al., 2015).

**Figure 1:**
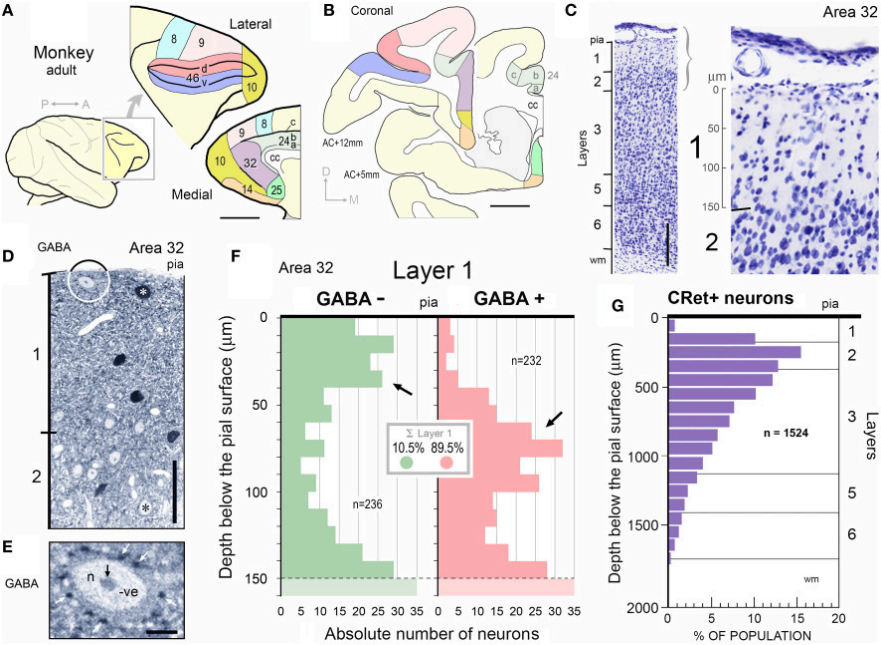
**(A)** Diagram of the adult monkey brain highlighting cytoarchitectural areas on the lateral and medial surfaces. Note splayed principal sulcus on lateral surface. Scale bar: 1 cm. **(B)** Two representative coronal sections at +5 and +12 mm to anterior commissure (AC) showing location and extent of cortical areas in **(A)**. Dorsal, d; Medial, m. Corpus callosum, cc. Scale bar: 5 mm. **(C)** Coronal Nissl stained section of area 32 indicating cortical lamination. Scale bar: 250 μm. Right panel: Enlargment of layers 1/2 with 50 μm depth marker in layer 1. **(D)** Post-embedding GABA immunocytochemistry performed on a 1 μm semithin section through area 32. GABA+ neuron in layer 1 is indicated (white asterisk). GABA- neuron lying immediately beneath the pial surface is shown encircled (enlarged in **E**). A GABA- pyramidal-shaped profile in layer 2 is indicated (black asterisk). Scale bar: 50 μm. **(E)** Numerous dark GABA+ punctae (white arrows) are closely opposed to the soma of the subpial GABA- neuron highlighted in **(D)**. Note also GABA- profiles abutting the same soma. (Nucleus (n) and nucleolus (black arrow) are indicated). **(F)** Depth distribution histograms of GABA- (*n* = 236) and GABA+ (*n* = 232) neurons below the pial surface in area 32. Layer 1/2 boundary is indicated (dashed line). **(G)** Histogram showing the percentage distribution of CRet+ neurons below the pial surface in area 32. (Sample population *n* = 1524 CRet+ neurons). Laminar boundaries are indicated.

Layer 1 contains a numerically small population of excitatory and inhibitory cells — most, possibly all, being local circuit neurons (LCNs). In the medial prefrontal cortex (mPFC) of adult macaque monkeys, layer 1 has approximately 560 neurons under 1 mm^2^ of pial surface — less than 0.5% of the total number of neurons in a column of cortex spanning layers 1–6 (Figures 1A–C; Gabbott and Bacon, 1996a,b). LCNs containing the inhibitory neurotransmitter gamma-aminobutyric acid (GABA) represent about 90% of neurons in layer 1 and are predominantly situated mid-lamina, whereas the GABA immunonegative (-) LCNs (presumed excitatory cells), which account for ~10% of layer 1 neurons, are mainly located directly beneath the pia and toward the boundary with layer 2 (Figures 1D–F).

Evidence indicates that LCNs in developing layer 1 can be fractionated by structure and function, and by genetic and molecular markers (DeFelipe et al., 2013; Muralidhar et al., 2014; Lee et al., 2015; Varga et al., 2015). Cajal-Retzius cells are excitatory LCNs recognized early in cortical development by their expression of the calcium binding protein calretinin (CRet; Glezer et al., 1992; Weisenhorn et al., 1994; Yan et al., 1995a,b; Frassoni et al., 1998; Ulfig, 2002; Barinka and Druga, 2010; Schwaller, 2014; Girard et al., 2015) and the secretion of the extracellular matrix glycoprotein reelin (Del Río et al., 1995; Derer et al., 2001; Abraham et al., 2005; Meyer, 2010). The secretion of reelin by Cajal-Retzius cells (and other neurons) plays an important role in choreographing the developmental blueprint of radial cell migration, laminar and columnar differentiation, as well as the formation and plasticity of synaptic circuitry during cortical maturation (Frotscher, 1998, 2010; Nishikawa et al., 2002; Fatemi, 2008; Meyer, 2010; González-Gómez and Meyer, 2014; Lee et al., 2014; Ramos-Moreno and Clascá, 2014; Chai et al., 2015; Varga et al., 2015).

CRet immunopositive (+) neurons, including Cajal-Retzius cells, are present in layer 1 throughout corticogenesis. In humans and monkeys, a subset (or subsets) of CRet+ Cajal-Retzius cells are known to persist into adulthood, greatly reduced in numbers and with altered morphologies (Condé et al., 1994; Belichenko et al., 1995; Del Rio et al., 1996; Del Río et al., 1997; Gabbott and Bacon, 1996a; Gabbott et al., 1997; Martín et al., 1999; Gil et al., 2014; González-Gómez and Meyer, 2014). In the adult monkey PFC, CRet+ neurons constitute c.16% of the total number of neurons in layer 1 (Gabbott and Bacon, 1996b). This CRet+ population is composed of both GABA+ LCNs (c.83%) and GABA- cells (c.17%) (Gabbott and Bacon, 1996b, and this study; see also Yan et al., 1995a; Miettinen et al., 1997; Melchitzky et al., 2005).

Compared with other cortical LCNs, the morphology and synaptic connectivity of CRet+ layer 1 neurons during development and persisting into adulthood remain understudied — especially in primates (Del Rio and DeFelipe, 1997; Meskenaite, 1997; Džaja et al., 2014). The detailed neuroanatomical research described here uses archived material to classify the morphological varieties of CRet+ neurons (including persisting Cajal-Retzius cells) in layer 1 of adult macaque monkey PFC. With this comparative foundation, the study then concentrates on a unique class of CRet+/GABA- layer 1 neuron, present in adulthood. The characteristic features of this exceptional neuronal phenotype are its location directly beneath the pial surface and a smooth unipolar soma with a thick primary dendrite that gives rise to a horizontal fan-like dividing arbor. As a result of the latter structural hallmark these distinctive CRet+ neurons have been named “subpial fan (SPF) cells.”

By using correlated light and electron microscopy, the synaptic input to identified SPF cell dendrites is defined. Furthermore, data are presented detailing the spatial distribution of SPF varicose axonal arbors and the ultrastructural identity of local and distant post-synaptic target structures. In addition to SPF cells with normal structural integrity, degenerating CRet+ neurons (including SPF cells) were present in layer 1 — for completeness, an account of SPF cells undergoing degeneration is also given.

The observations are related to the structure and function of other LCN neuron types and afferent systems ramifying in layer 1 — expressly their relation with the Cajal-Retzius cell family. Although previous studies highlight the structural complexity of layer 1 neurons in primates (Meyer et al., 1999; Rakic and Zečević, 2003) consideration is given, where appropriate, to studies in lower mammals. Also considered is the possible structural and functional relationship of SPF cells with the apical dendritic tufts in layer 1 derived from pyramidal cell minicolumns (Marín-Padilla, 1984, 2015; Gabbott, 2003).

While the study has exclusively used CRet to identify layer 1 LCN subtypes, it provides new and important information about the morphology and synaptic connectivity of SPF cells — which contribute to the function of this lamina in the PFC of a primate species. Of significance, primate dorsolateral (dl) PFC is involved in the executive control of goal directed behaviors, whereas mPFC mediates emotional and autonomic functions (Vogt, 2009; Passingham and Wise, 2012). Moreover, evidence suggests that abberations in the maturation of PFC underlie specific neurodevelopmental disorders and psychiatric conditions in humans (Iafrati et al., 2014; Schubert et al., 2015).

## Materials and methods

### Subjects and experimental methods

Post-mortem brain tissue for this study was obtained from Professor Kevan Martin and Dr John Anderson (Oxford/Zurich), Professor Alan Cowey (Oxford) and Professor Wolfram Schultz (Cambridge). The tissue came from eight normal young adult/adult monkeys (*Macaca fasicularis*; 5 male and 3 female; 4–12 years of age). Several of the cases had been used in experiments studying the monkey visual system and in experiments exploring reward mechanisms in the brain. All surgical and related procedures were conducted in accordance with the Society for Neuroscience “Policy on the use of animals in neuroscience research” and were licensed separately at the Universities of Oxford and Cambridge under the U.K. Animals (Scientific Procedures) Act, 1986.

At the end of these experiments monkeys had been given lethal overdoses of anesthetic. When sufficiently anesthetized animals were transcardially perfused initially with 0.9% physiological saline and then with either 4% paraformaldehyde alone or with 3% paraformaldehyde and 0.5–2.0% glutaraldehyde (Taab, Reading, UK) in 0.1M phosphate buffer (pH 7.4) at room temperature.

### CRet immunocytochemistry

Blocks of tissue containing mPFC/cingulate cortex (BAs 25, 32, 24a,b,c) and dlPFC (BAs 9, 46) were carefully excised from both brain hemispheres (Figures 1A,B; Paxinos et al., 2000). These tissue blocks were sectioned serially (80, 100, 200, or 250 μm thickness) using a Vibraslice microtome (Campden Instruments, Loughborough, UK) in the coronal plane or tangential to the pial surface. Sections were then rinsed (3 × 15 min) in 50 mM TRIS-HCl pH 7.4 buffer (TRIS). Further processing was undertaken on free-floating sections.

Selected sets of serial coronal sections, together with tangential sections through the uppermost layers of the cortex, were rinsed in TRIS buffer containing 0.5% Triton X-100 for 1–2 h or were freeze-thawed. Some sections were treated in a microwave at 600 W for 60–90 s in TRIS buffer pH 6.0. Sections were subsequently washed in 20% normal goat serum [30–60 min diluted in TRIS buffer pH 7.4 at room temperature (RT)] and incubated in a primary polyclonal antiserum against calretinin [Code 7696; SWant raised in rabbit (Schwaller et al., 1993); dilution 1000–2500 with 0.01% NaN_3_] for 2–3 days at 4°C or overnight at RT.

Immunolabeling was visualized with standard immunoperoxidase procedures using a species matched Vectastain ABC kit (Vector laboratories, Peterborough) and developed with either: (i) 3,3′-diaminobenzidine tetrahydrochloride (DAB) as chromogen [incubating sections in TRIS (pH 7.4) with 0.05% DAB (Sigma) and 0.01% H_2_O_2_ for 3–10 min at RT], or (ii) using the SG (slate gray) Vectastain peroxidase substrate kit (SK-4700; Vector laboratories, Peterborough) by incubating sections for 3–8 min at RT. Specific immunolabeling was absent from sections incubated without primary anti-serum or peroxidase linked antibody. (Importantly, the dark yellow lipofuscin-rich profiles referred to below were seen in the untreated tissue sections *prior* to immunocytochemistry and exposure to osmium tetroxide — see **Figures 4I–K**, **13E,G,I**, **14B,D**). Sections for light microscopy alone were mounted in series on glass slides, air-dried, dehydrated in alcohol, passed through xylene, and embedded in DePeX mountant then coverslipped.

Archived Nissl stained sections were used to define the areal and laminar cytoarchitecture of frontal cortices (Figures 1A–C; Carmichael and Price, 1994; Saleem et al., 2013).

Sections for correlated light and electron microscopical study, were first treated with osmium tetroxide [1% OsO_4_ (aq), 45 min], dehydrated in alcohols (with the 70% alcohol containing 1% uranyl acetate), passed through absolute alcohol, swiftly through propylene oxide, and embedded in Durcupan resin (ACM Fluka). Finally, sections were flat-embedded, coverslipped and cured at 60°C for 48 h.

### Light-microscopy

Sections were initially examined in a light microscope and structures of interest recorded with through-focus photomicrographs, digital images, and drawings.

#### Dendritic morphometry

**Figure 2:**
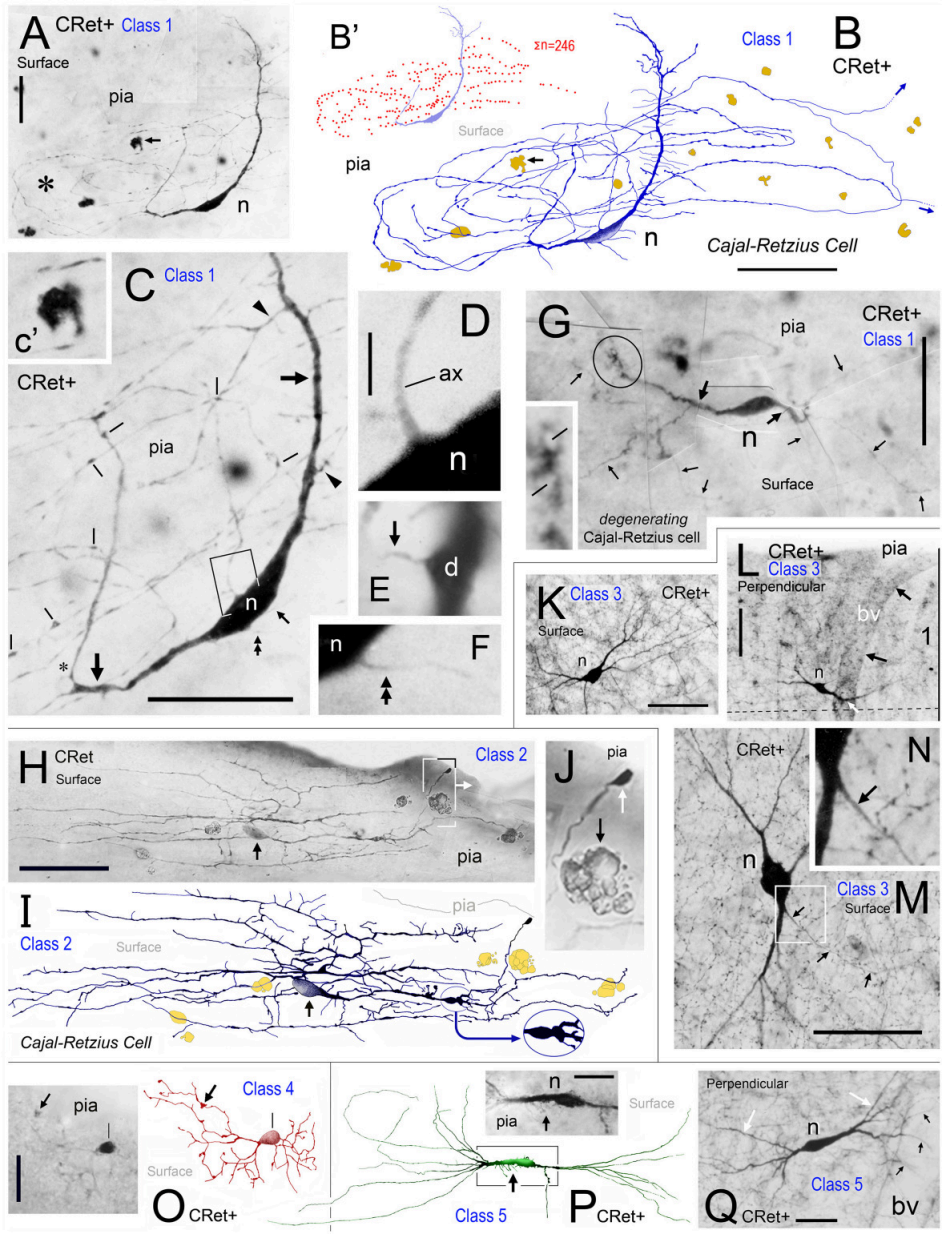
**Calretinin immunopositive (CRet+) neurons in layer 1 of monkey PFC — Classes 1–5. (A)** Class 1. Area 32. Surface view of a superficial horizontal fusiform CRet+ neuron (n) lying immediately below the pial surface. Note the widespread distribution of labeled processes which ramified throughout the depth of layer 1 (asterisk). A deposit of cellular debris (non-immunolabeled) is indicated (small arrow). Montaged photograph. Scale bar: 25 μm. **(B)** Reconstruction of the CRet+ neuron seen in **(A)**. Thick dendrites emerge from opposite ends of the highly fusiform soma. Numerous thin varicose processes arise from dendrites and ramify radially to form a swirling varicose plexus in the vicinity of the soma and its main dendrites. Two labeled processes that course distally before being truncated (small arrows). This neuron is immunomorphologically identified as a Cajal-Retzius cell. Scale bar: 50 μm. **(B**′**)** Distribution of individual axonal swellings (red dots) along the immunolabeled processes of the cell seen in **(A,B)**. Scale bar: 50 μm. **(C)** Detail of the perisomatic region of the CRet+ neuron in **(A)**. Note the thick primary dendritic processes (arrows) and the numerous side shoots (triangles). Varicosities/swellings along the labeled processes are indicated (lines). Scale bar: 40 μm. **(C**′**)** High power image of the cellular debris indicated in **(A). (D)** Enlargement of the boxed region in **(C)** showing a main axon-like process (ax) arising from the labeled soma (n). Scale bar: 5 μm. **(E)** Labeled thin caliber process (arrow) arising from a main dendritic process **(D)**. **(F)** Labeled thin process (double headed arrow; see **C**) emerging from the soma (n). **(G)** Class 1. Area 24b. Surface view of a degenerating bipolar CRet+ neuron (n) in upper layer 1 with dysmorphic dendrites and processes (thick arrows). Vacuoles are present in a segment of one primary dendrite (encircled and shown enlarged in inset). Labeled thin collateral processes arising from the main dendrites are indicated (thin arrows). This neuron has the morphological hallmarks of a Cajal-Retzius cell. Scale bar: 60 μm. **(H)** Class 2. Area 32. A horizontal multipolar CRet+ neuron with an expansive elongated dendritic arbor in upper layer 1. Cell body (arrow). Highlighted regions shown in **(I,J)**, respectively. Note the clumps of lipofuscin-rich debris (see **J**). (Oblique view). Scale bar: 100 μm. **(I)** Reconstruction of the CRet+ cell in **(H)**. The parent dendrites bifurcate into long daughter segments forming an elongated dendritic arbor. Large varicosities along dendrites (oval inset) and the labeled process with a subpial terminal expansion (boxed region) are highlighted. (Clumps of lipofuscin-rich debris shown in dark yellow). This cell has the morphological hallmarks of a Cajal-Retzius cell. **(J)** Enlarged image showing the subpial terminal expansion (white arrow) and a clump of lipofuscin-rich debris (black arrow) in **(H). (K)** Class 3. Area 9. Surface view of a standard multipolar CRet+ cell (n) in mid-layer 1. Scale bar: 50 μm. **(L)** Class 3. Area 46d. Perpendicular view of a standard multipolar CRet+ cell (n) in lower layer 1 near the border with layer 2 (dashed line). Note proximally bifurcating dendritic trees. A blood vessel (bv) is seen descending perpendicularly through the cortex (black arrows). One of the CRet+ labeled dendrites sends branches around the vessel (white arrow). Scale bar: 50 μm. **(M)** Class 3. Area 32. Surface view of a standard multipolar CRet+ neuron (n) in mid/lower layer 1. Note axonal process (arrows). Scale bar: 50 μm. **(N)** Initial segment of the axon from neuron in M. **(O)** Class 4. Area 25. A small-sized neurogliaform CRet+ neuron in upper layer 1. Note dense plexus of immunolabeled dendrites (arrow). Scale bar: 25 μm. **(P)** Class 5. Area 46v. Surface view of a spindle-shaped CRet+ neuron in upper layer 1 with a tuft of dendrites originating from opposite poles of the soma. Inset: numerous fine fibers emerging from the labeled soma (arrow). Scale bar: 20 μm**. (Q)** Class 5. Area 24b. Perpendicular view of a spindle-shaped CRet+ neuron in mid-layer 1 with tufts of dendrites (white arrows). Several dendrites (black arrows) wrap around a neighboring blood vessel (bv). Scale bar: 20 μm.

**Figure 3:**
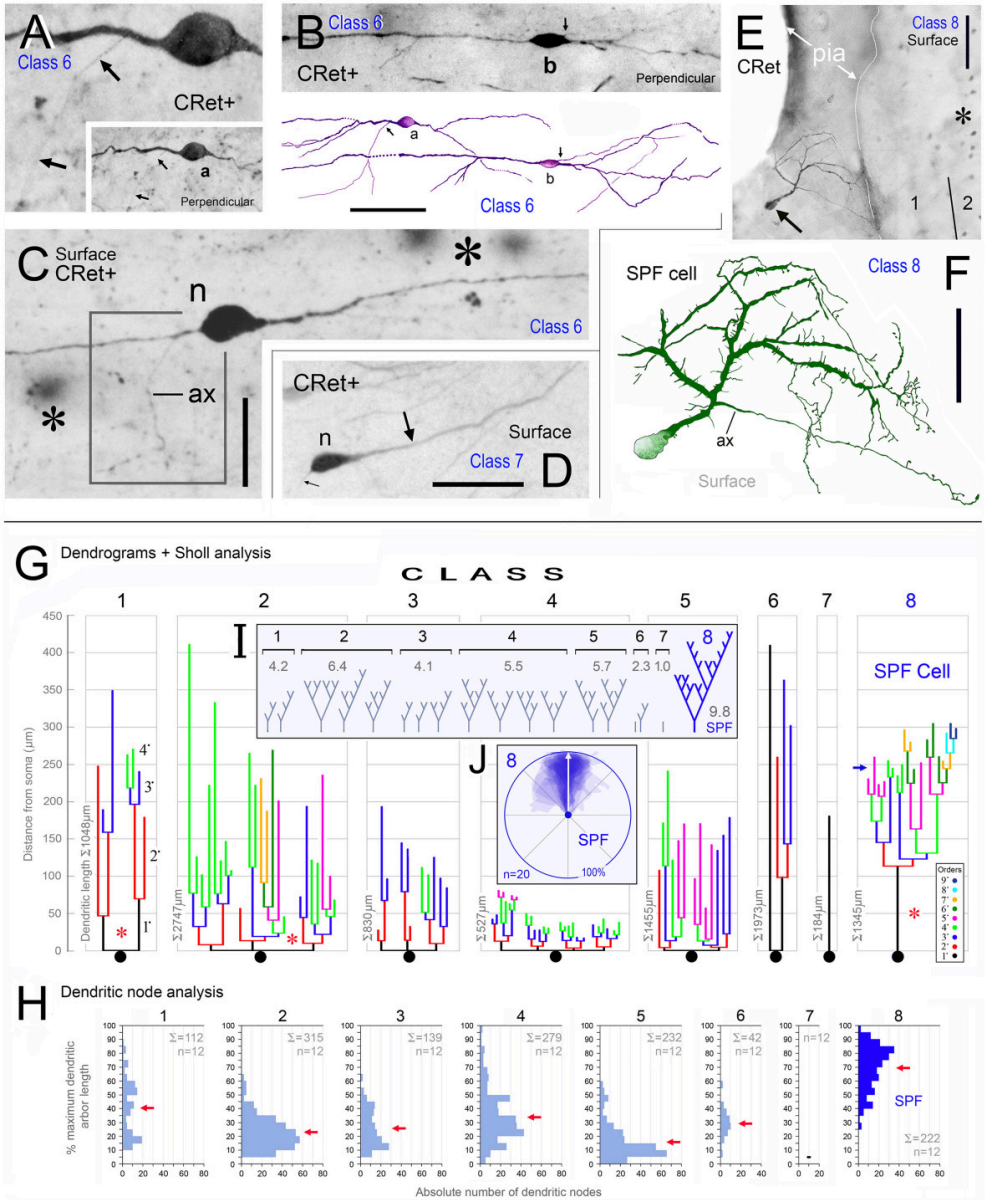
**Calretinin immunopositive (CRet+) neurons in layer 1 of monkey PFC — Classes 6–8 (A–F). (A,B)** Class 6. Perpendicular views of two deep horizontal bipolar CRet+ neurons (a, area 25; b, area 9) in lower layer 1. Fine axonal processes are indicated (arrows). Composite drawing of cells a and b. Scale bar: 100 μm. **(C)** Class 6. Area 24c. Surface view showing a deep horizontal bipolar CRet+ neuron in lower layer 1. Profiles of CRet+ neurons in upper layer 2 (lying deeper in the section) are seen (asterisks). (Axon, ax). Scale bar: 50 μm. **(D)** Class 7. Area 24c. Simple unipolar CRet+ neuron (n). (Dendrite arrowed). Scale bar: 50 μm. **(E)** Class 8. Area 46d. CRet+ SPF cell (arrow). CRet+ neurons in layer 2 (asterisk). Pial surface is indicated (oblique view). Scale bar: 50 μm. **(F)** Reconstruction of the cell in **(D)**. Note unipolar soma and elaborate fan-like arrangement of higher order dendrites which give rise to numerous thorns. An axon arises proximally along the primary dendrite. Scale bar: 50 μm. Morphometric analysis of dendritic arbors **(G–J). (G)** Dendrograms for the eight classes of CRet+ neurons in layer 1 of adult monkey showing the lengths, nodal points. and main dendritic segments (filamentous and thorn processes are not indicated, asterisks). A single representative cell in each class is shown. **(H)** Bar charts of the distribution (absolute number) of dendritic node locations as a function of maximum dendritic arbor length (100%). [Data from 12 cells in each class are presented. Total absolute numbers of nodes (Σ) are given]. Note the highly characteristic distribution of nodes for class 8 SPF cells. (Means of distributions, red arrows). **(I)** Dendritic tree topologies of the 8 CRet+ cell classes presented in H together with the corresponding WeST values (1.0–9.8). **(J)** Outlines of 20 SPF cell dendritic boundaries superimposed over their major caliper axes. Somata centroids act as root points.

**Figure 4:**
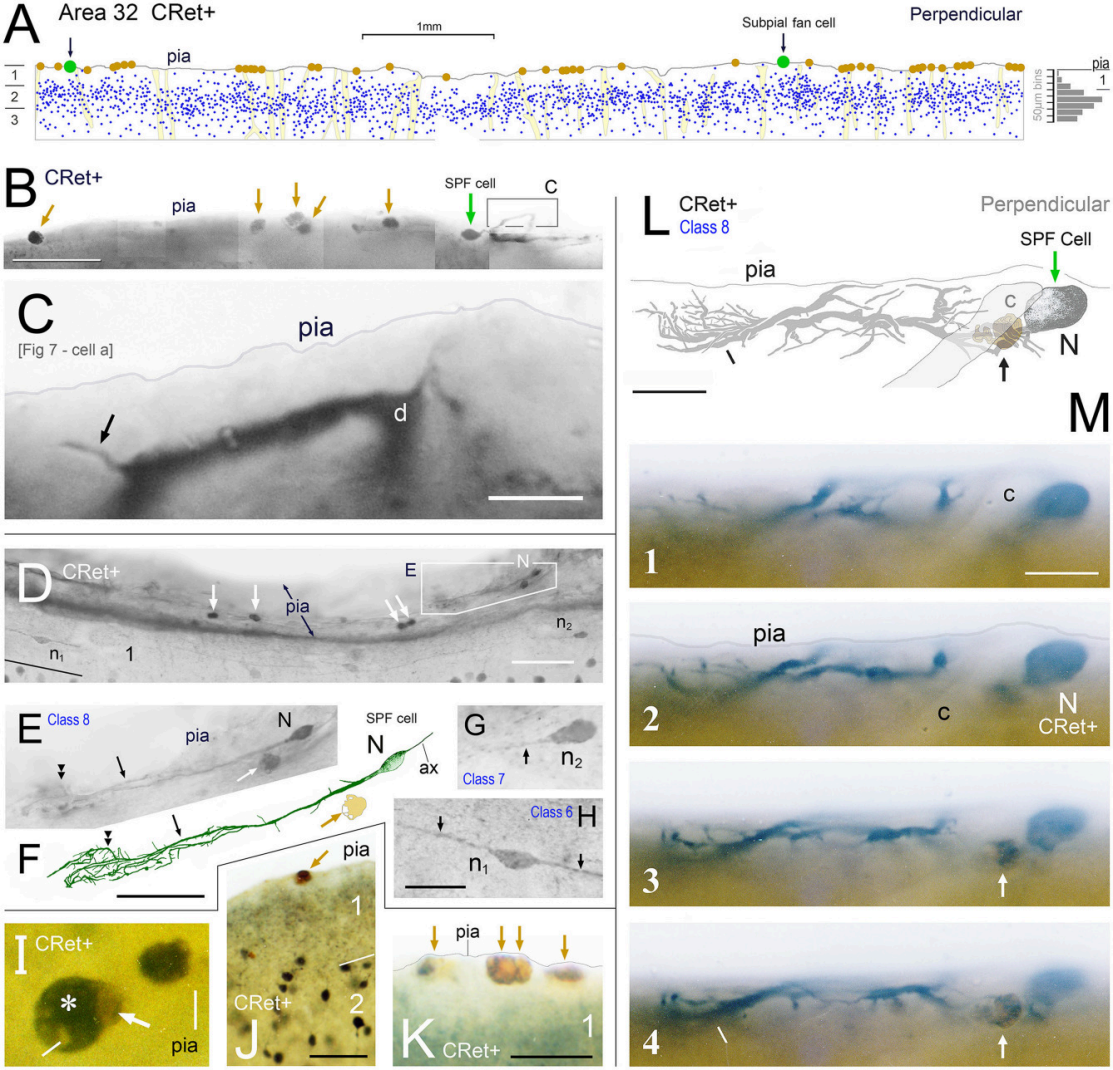
**(A)** Drawing of a perpendicular coronal strip through area 32 showing the location of CRet+ neurons (blue dots) in layers 1, 2, and upper 3. Subpial thorn (SPF) cells (green dots). Subpial somatic profiles containing dark yellow lipofuscin (brown dots). Profiles of penetrating blood vessels (yellow). A summary depth-frequency distribution of CRet+ cells is given on the right. Scale bar: 1 mm. **(B)** Photographic montage of the pial surface of a coronal section through area 46v. A SPF cell is indicated with part of its dendritic arbor highlighted. Note discrete dark cellular profiles (brown arrows). Scale bar: 100 μm. **(C)** Part of the main immunolabeled dendrite (d) of the SPF cell in **(B)**. A dendritic thorn is indicated (arrow). Scale bar: 10 μm. [The morphology of this SPF cell is shown in **Figure 7**–cell a]. **(D)** Oblique view of the pial surface in area 24b showing a CRet+ SPF cell (N) and numerous dark somatic profiles containing cellular debris distributed under the pia (white arrows). Note also two CRet+ cells in mid-layer 1. Scale bar: 100 μm. **(E)** Class 8. Enlargement of the SPF cell in **(D)**. Scale bar: 100 μm. **(F)** Drawing of the dendritic arbor of the SPF in **(D,E)**. Axon, ax. (Subpial lipofuscin-rich profile — brown). Scale bar: 50 μm. **(G,H)** Magnified views of CRet+ cells n_1_ and n_2_ in **(D)**. Cell n_1_ is a deep horizontal bipolar cell (Class 6) and n_2_ a simple unipolar neuron (Class 7). Dendrites (arrows). Scale bar: 20 μm. **(I)** View of pial surface showing a vacuolated subpial CRet+ profile (gray immunolabeling — asterisk) with a large brown swelling (white arrow). Scale bar: 10 μm. **(J)** Area 25. Large brown subpial profile (arrow). Scale bar: 100 μm. **(K)** Cluster of brown subpial profiles in area 9. Scale bar: 50 μm. **(L)** Perpendicular view of a SPF cell (N) in area 46v. The outline of a descending capillary is indicated (c). Cellular debris containing lipofuscin (arrow). Terminal dendritic plexus (line). Scale bar: 20 μm. **(M)** Series of micrographs (1–4) taken at various focal planes through tissue section containing the CRet+ SPF cell indicated in **(L)**. Capillary (c); deposit of cellular debris associated with labeled dendrite (arrow); and terminal dendritic plexus (line). Scale bar: 20 μm.

Eight distinct classes of CRet+ neuron in layer 1 were identified qualatively on the basis of dendritic architecture (Figures 2–**4**). Quantitative analyses were then used to provide morphometric parameters indexing each class and to test whether SPF cells represented a distinct type of CRet+ cell. Accordingly, the dendritic arbors of 12 well-labeled neurons per class were reconstructed in three dimensions (3D) using a Neurolucida/Neuroexplorer (MicroBrightField, USA) computer system attached to a Leitz light microscope. Arbors were assessed quantitatively using the following procedures: (i) construction of dendrograms (Uylings and Van Pelt, 2002); (ii) Sholl analyses; (iii) distributions of dendritic branch points with respect to the maximum (100%) arbor length; and (iv) a “Weighted-Segment/Tip” (WeST) analysis. This index reflects, to a first approximation, the overall structural topology of a LCN dendritic arbor — composed of one or more binary dendritic trees. Each segment in a dendritic tree was given the value of its centrifugal order (i.e., primary dendrite (root segment) = 1, secondary dendrite = 2, tertiary dendrite = 3, etc). The total weighted values for all segments in a single dendritic tree were calculated then divided by the number of terminal dendritic tips in the tree. A mean WeST value was calculated for all the dendritic trees from one neuron and averaged across cells (*n* = 10) from the same class. Significant interclass differences were assessed using ANOVAs followed by multiple *post*-*hoc* Bonferroni *t*-tests (significance *p* < 0.05).

#### 3-D reconstruction/rotation of SPF cells

**Figure 5:**
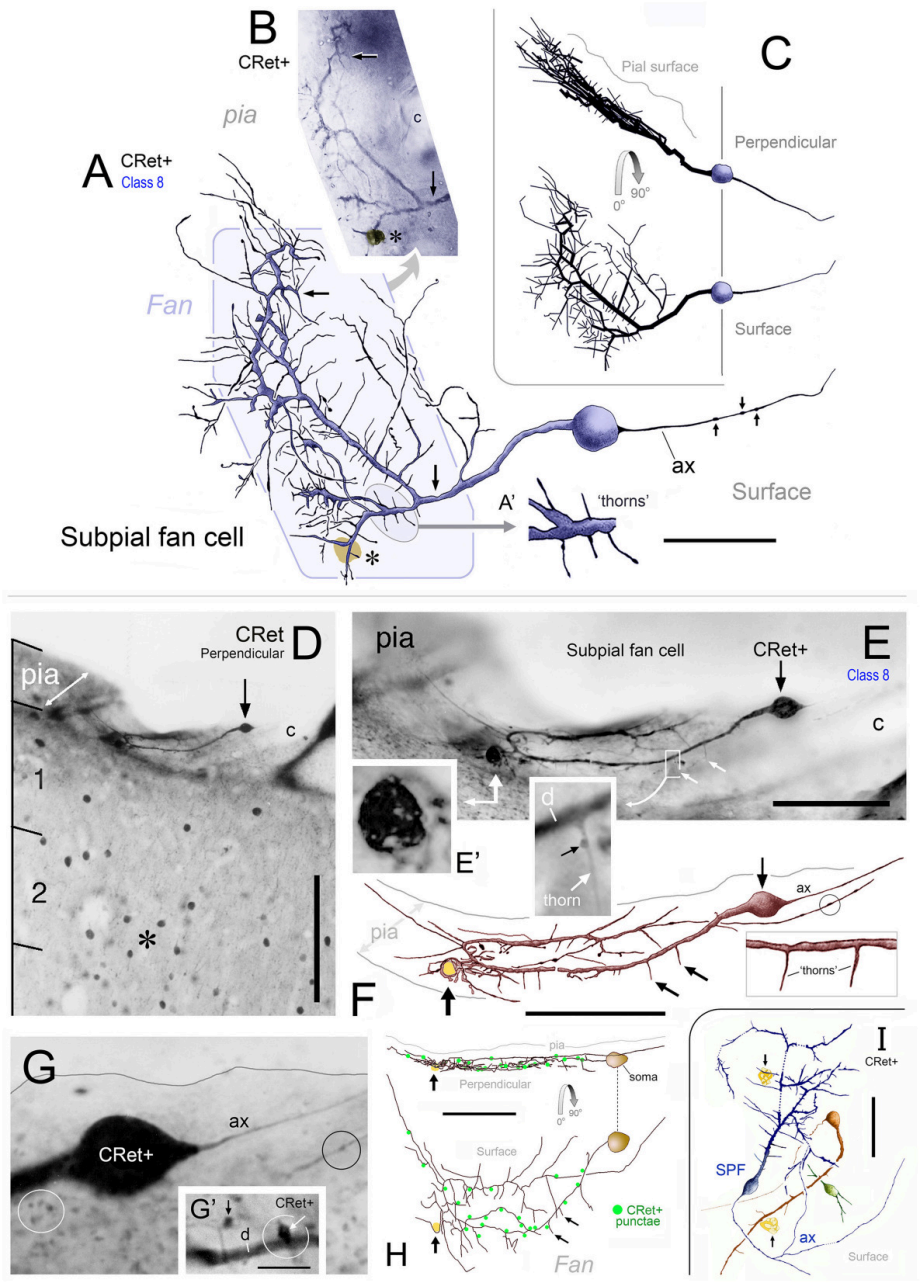
**(A)** Class 8. Area 46d. Reconstruction of a CRet+ SPF cell. Note the radiate fan-like distribution of the dendritic processes. Numerous thorn-like protrusions emanate from the main processes. Swellings along the axonal process (ax) are indicated. Scale bar: 50 μm. Inset **(A**′**)**: Enlarged image of the region in **(A)** illustrating several long, fine thorn-like protrusions arising from a thick secondary dendrite. **(B)** Photomicrographic montage of the dendritic arbor indicated by the polygonal region in **(A). (C)** 3D rotations showing the distribution of the cellular processes viewed in the plane perpendicular to the pial surface (90°) and viewed parallel with the pial surface (0°). **(D)** Class 8. Area 24b. CRet immunolabeling in superficial layers 1–2. A CRet+ SPF cell is indicated (arrow). Note the paucity of other CRet+ neurons in layer 1 compared with the increased density of labeled neuronal somata in layer 2 (asterisk). The slanted white double headed arrow indicates the plane of the pial surface which lies oblique to the axis of viewing. Capillary, c. Scale bar: 200 μm. **(E)** Enlarged image of the SPF cell in **(H)**. One pole of the ellipsoid soma (arrow) gives rise to a thick branching dendrite that recurves and courses tangentially directly beneath the pial surface. Thorn-like processes arising proximally from the main dendrite are indicated (small white arrows). Region outlined is enlarged in inset which shows a CRet+ puncta abutting a dendritic thorn (d, dendrite). A dark vacuolated cellular profile is shown enlarged in **(E**′**)** Capillary, c. Scale bar: 100 μm. Inset shows **(F)** Reconstruction of the neuron in **(E)** (arrow). Fine thorn-like processes (small arrows) emanate from the thick main process (see inset). A small-caliber terminal process gives rise to several varicosities (one of which is encircled). Scale bar: 100 μm **(G)** Photomicrograph of the SPF soma — note lack of filaments arising from soma (c.f. Figures 2C,F). A thin axon-like processes (ax) emerges from one pole of the soma. Swelling along an immunopositive process (black circle). CRet+ punctae (presumed synaptic boutons) are present in the neighboring neuropil (white circle). Scale bar: 25 μm. **(G**′**)** A CRet+ axonal swelling (white arrow) closely opposed to a CRet+ distal dendritic process (d). Another CRet+ puncta is indicated (black arrow). **(H)** 3D-rotations of the SPF cell to show the distribution of the immunolabeled cellular processes when viewed in the perpendicular plane and in surface view. Note the fan-like spread of the labeled processes. Indicated are the visible CRet+ punctae (green dots) abutting the processes of the SPF cell. Scale bar: 100 μm. **(I)** Surface view of a group of three neighboring CRet+ neurons in upper layer 1 of area 46d. Categorized as a class 8 SPF cell, a superficial class 7 cell and the profile of a partly immunolabeled neuron. Clumps of lipofuscin (arrows) (Axon, ax). Scale bar: 100 μm.

The dendritic and axonal arbors of 15 SPF cells were reconstructed in 3D. The locations of varicosities (putative synaptic boutons) along SPF cell axons were recorded in the 3D data sets — likewise the positions of CRet+ punctae in close association with identified SPF cell dendrites (**Figures 5E,G′**). Neurons were subsequently rotated and viewed either in the *perpendicular* plane (90°) or in *surface* view (0°) with respect to the pia (see lower left inset in **Figure 7**).

The tangential boundary distributions of the SPF cell dendritic arbors were assessed by calculating their major caliper axis. Arbors were then superimposed along the major axis with somatic centroids as root points. Arbor boundaries were transformed so that their maximal extents along the major axis were the same (100%) — providing an overall comparison of SPF cell dendritic arbor polarization (Figure 3J).

#### Spine densities of SPF cell dendrites

Linear spine densities (number of spines per micron) along spine bearing primary dendritic segments (lengths >50 μm) selected from at least five CRet+ cells per class were calculated as described previously (Gabbott et al., 1995).

#### Areal density of axonal varicosities — “Local” vs. “Distant” zones

Preliminary analysis of SPF axons (*n* = 8; from areas 24b, 32, 46, 9) in the tangential plane indicated a variation in the clustering of identified axonal swellings with respect to distance from parent somata (**Figure 8**). To assess this quantitatively, individual axonal arbors were divided into two separate concentric regions — “local” (within the vicinity of the soma) and “distant” zones (**Figure 8**). A general guideline was established that the division between the two zones occurred between 150 and 200 μm radius from the parent soma (see **Figures 8D,E**). This guideline was adjusted to accommodate individual axonal arbors (**Figures 8A,C**).

Axonal swelling density per unit area of cortical surface was then calculated (c.f. linear density per unit axon length) for each zone within an individual arbor (**Figure 8**). Thirty counting quadrats (optimized at 50 × 50 μm) were positioned randomly by computer within *each* local and distant zone of the arbor. A “test” quadrat was defined as lying entirely within the respective zone and containing an axonal segment from the identified SPF cell with at least one varicosity. The total number of identified swellings in each test quadrat were then counted (with upper/left inclusion borders and lower/right exclusion borders; Gabbott and Somogyi, 1986).

Mean “local” and “distant” axonal varicosity areal densities were calculated per arbor and then for the total sample of the eight arbors. Overall mean “local” and “distant” data were compared statistically using a two-tailed Student *t*-test (significance *p* < 0.05).

Finally, an optimized lattice (divided into 20 × 20 μm squares) was used to visualize the tangential density distribution of axonal swellings from individual SPF cells. The relative frequency of varicosities in each square was derived and contour plots created using Surfer 7 mapping software (Golden Software, Colorado. USA).

### Ultrastructural analysis

Selected well-labeled SPF neurons (*n* = 12) were examined ultrastructurally using correlated light and electron microscopy (Gabbott et al., 2012). In brief, long series of serial ultrathin sections were cut through identified neural structures seen in the light microscope, collected on Formvar-coated single slot grids, stained with Reynold's lead citrate and subsequently examined in an electron microscope (Gabbott et al., 2012).

#### Synaptic input to identified SPF cells

The somata, dendrites, thorns, filamentous processes, and spines of SPF cells were traced through serial sections to identify asymmetric (A-type) and symmetric (S-type) synaptic inputs (Peters et al., 1991). The CRet+ and CRet- nature of presynaptic boutons was noted. Specific structures were reformed from ultrathin section profiles using Reconstruct (Fiala, 2005).

#### Synaptic output of identified SPF cells

Post-synaptic target structures were categorized as either dendritic spine (sp) or shaft (sh), cell body (cb), axon initial segment (ais), or unclassifiable due to lack of structural criteria in the post-synaptic element (?). A selected sample of local (*n* = 74) and distant (*n* = 79) boutons (with clearly identified synaptic junctions) were analyzed from the axonal arbors of five representative SPF cells (areas 9, 24b, 25, 32, and 46d) in four animals. Possible differences in the distribution of the cellular compartments innervated by the “local” and “distant” axonal arbors were assessed. In addition, each compartment category was subdivided into CRet+ or CRet- post-synaptic targets.

Electron micrographs of identified structures were produced photographically, scanned and digital image files created.

### Combined pre-embedding CRet and post-embedding GABA immunocytochemistry

Coronal and parasagittal tissue sections (from two animals fixed with high glutaraldehyde content) were reacted immunocytochemically for CRet using the pre-embedding immunocytochemical method described above — with Vector SG as peroxidase substrate. Selected sections were then embedded in resin. Serial semithin 0.5–2 μm thick resin sections were cut through the processes and somata of identified CRet+ SPF cells (from areas 32, 24, and 46v) and subsequently reacted for GABA using post-embedding immunocytochemistry — with DAB as peroxidase substrate (Gabbott and Bacon, 1996a). The GABA immunoreactive nature of identified CRet+ SPF cells (*n* = 7) was then assessed by the detection of intense dark-brown GABA immunolabeling within their SG immunolabeled somatic/nuclear profiles. [Note: SG reaction product in CRet+ immunolabeled neurons does not prevent subsequent detection of GABA immunolabeling in the same material visualized with DAB]. The material was also used to assess the presence of GABA immunoreactivity in 100 CRet+ neurons throughout layer 1 in area 32 — thereby allowing the ratio of CRet+/GABA- to CRet+/GABA+ neurons to be derived.

The depth distributions of GABA immunonegative (GABA-) and GABA immunopositive (GABA+) nuclear profiles through layer 1 and into the upper part of layer 2 (Figure 1F) were also determined. The number of GABA+ and GABA- nuclear profiles in a sampling strip (250 μm wide), divided vertically into twenty 10 μm deep sampling tiers, were counted. (The left and bottom borders of each tier represented exclusion boundaries — Gabbott and Somogyi, 1986). A sufficient number of randomly positioned sampling strips (*n* = 10 in each animal; right hemisphere) were used to compile depth distribution histograms for equal numbers (*n* = 200) of GABA+ and GABA- nuclear profiles (Figures 1D–F).

### Illustrations

All image files were finally imported into Adobe Photoshop© CS6, adjustments made for contrast and brightness, photographic montages then assembled and composite illustrations prepared.

## Results

### CRet+ immunoreactivity throughout prefrontal cortex

Specific CRet+ immunolabeling was present throughout layers 1–6 of the PFC areas studied and in the underlying white matter. The morphologies and quantitative laminar distributions of calretinin immunopositive (CRet+) neurons in macaque PFC have been previously described (Condé et al., 1994; Gabbott and Bacon, 1996a,b; Gabbott et al., 1997). The peak distributions of CRet+ neurons in primate PFC was in layer 2 with a small fraction (< 1%) of the total CRet+ population present in layer 1 (Figure 1G — area 32; Condé et al., 1994; Gabbott and Bacon, 1996b).

### Classification of layer 1 CRet+ cells

Several classes of CRet+ neurons were immunolabeled throughout the depth of layer 1 and were present across all PFC areas (Figures 2, 3A–F, 4D,G,H). The classification starts with bi- and multipolar cells and proceeds to unipolar cells. The term “superficial” refers to neurons in upper layer 1 while “deep” applies to cells in mid/lower layer 1.

Eight main classes were identified. These consisted of:

1. *Superficial horizontal fusiform cells* in upper layer 1 with large-sized highly ovoid somata (diameters of area equivalent circles: d.circ = 20–35 μm; max/min caliper diameter ratio = 4.6 ± 0.4; mean ± SEM, *n* = 18). Somata commonly gave rise to two (infrequently three) thick primary dendrites from opposite poles. These dendrites coursed parallel with the pia and issued long horizontal axon-like processes with numerous swellings (Figures 2A–F). Axon-like fibers also emerged from somata (Figures 2C,F). Classical axons arose from the soma and/or branch points along primary dendrites — they ramified extensively in the vicinity of the parent somata. Distal axonal fibers were truncated (Figure 2B);
2. *Superficial horizontal multipolar cells* with medium/large-sized somata (d.circ = 15–30 μm) located in upper layer 1 (Figures 2H,I). The primary dendritic processes (3–6 in number) lay parallel to the pia. They formed interwoven trees that were highly elongated (c.500+ μm length; c.200+ μm width; Figures 2H,I). The primary segments had frequent expansions along their lengths (Figure 2I). Numerous fine-caliber processes arose from dendritic stems and extended toward the pia giving rise to terminal bulbs abutting the pial surface (Figure 2J). One or more axon-like processes commonly emerged from cell bodies and/or from primary dendritic shafts — ramifying extensively both locally and distally throughout layer 1.
3. *Standard multipolar neurons* situated predominantly in the lower two-thirds of layer 1 (c. 50–150 μm below pia; Figure 1C) with fine axons that ramified locally in lower layer 1/upper layer 2 (Figures 2K–N). The dendrites had simple bifurcating patterns and could have low numbers of dendritic spines (0.47 ± 0.12 spines/micron; mean ± SEM, *n* = 5);
4. *Midget neurogliaform cells* located in upper layer 1 (Figure 2O). A small-sized multipolar neuron (d.circ = 5–10 μm) with fine-caliber radiate dendrites which branched frequently in the vicinity of the soma — numerous swellings were present along their lengths. The axons of these cells were not seen;
5. *Tufted cells*. Neurons with highly elongated somata (max/min caliper diameter ratio = 5.4 ± 0.6; mean ± SEM, *n* = 15: Figures 2P,Q) with horizontal tufts of long dendrites from opposite somatic poles. Some somata issued fine hair-like filaments, as shown in Figure 2P;
6. *Deep horizontal bipolar neurons* in mid/lower depths of layer 1 (Figures 3A–C). These cells had linear aspiny dendrites several hundreds of microns in length oriented horizontally — the axons arose from the cell body or proximal dendrite (Figures 3A–C, 4H);
7. *Simple unipolar neurons* aligned horizontally in layer 1. Most common in the lower two-thirds of layer 1 (c.50–150 μm below pia; Figure 1C). These cells possessed a single small/medium-caliber dendrite and a fine axonal process (Figures 3D, 4G, 5I). The somata of these neurons were smooth and dendrites could sometimes issue appendages (Figure 5I). (The unidendritic nature of these cells was not due to sectioning artifacts); and,
8. *Subpial fan (SPF) cells* are horizontal neurons located immediately beneath the pia (Figure 1C). They possessed unipolar somata from which arose a single stout primary dendrite that branched repeatedly into an arbor that fanned out across a plane parallel with the pial surface (Figures 3E,F). The morphology and synaptology of SPF cells are described in detail below.

It was not possible to derive a reliable estimate of the relative frequencies of each CRet+ cell class due to the truncation of dendrites which affected the classification of many immunolabeled cells. Nevertheless, pilot data across PFC suggest the following: neurons in classes 1, 2, and 8 *each* comprised less than 0.1% of the total CRet+ neurons population in layer 1; class 3 represented 30–50%; class 4, 0.5–1.5%; class 5, 1–4%; class 6, 40–60%; and class 7, 5–15%.

**Figure 6:**
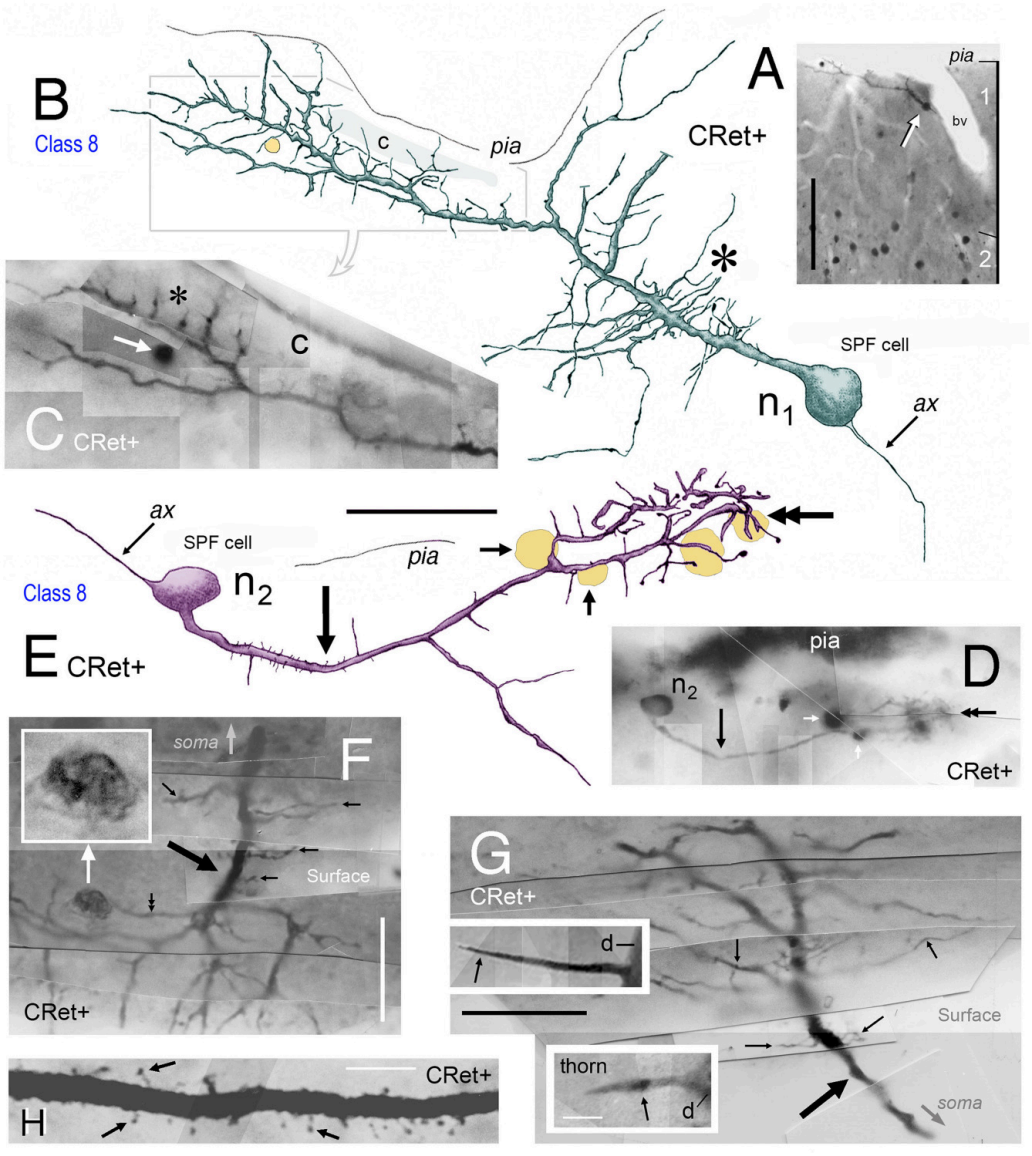
**(A)** Photomicrograph showing a class 8 CRet+ SPF cell (arrowed) in area 46v (oblique view). Numerous CRet+ neurons are present in layer 2. The SPF cell is closely associated with a penetrating blood vessel (bv). Scale bar: 200 μm. **(B)** Reconstruction of the SPFcell (n_1_) seen in **(A)** taken from three consecutive sections. Note the long fibrous processes associated with the primary dendrite (asterisk). A small-diameter penetrating capillary (c) is indicated. Axonal process (ax). Scale bar: 50 μm. **(C)** Photographic montage of the region indicated in **(B)**. Penetrating capillary (c). Thorns on dendritic processes (asterisk). A dark circular profile of cellular debris is indicated (white arrow). Scale bar: 25 μm. **(D)** Class 8. Photomicrograph of another CRet+ SPF cell (n_2_) in area 25. Dark profiles of cellular debris are indicated (white arrows). Scale bar: 25 μm. **(E)** Reconstruction of SPF cell (n_2_). Note the long primary process (arrow) and the terminal densely thorned plexus (doubled headed arrow). Axonal process (ax). Dark profiles of cellular debris as in **(D)**. Scale bar: 50 μm. **(F,G)** Photomicrographic montages showing fine processes (small arrows) arising perpendicularly from CRet+ labeled primary dendrites (thick arrows) from SPF cells in areas 24/32. In **(F)**, an irregular dark profile is associated with one process (double headed arrow; inset). Note the fan-like arrangement of processes in **(G)**. Scale bar: 25 μm. Insets in **(G)** show thorns on SPF cells. Dendritic stem, d. Scale bar: 25 μm. **(H)** Primary dendritic segment of a SPF cell in area 46. Numerous dendritic spines with different morphologies (arrows). Scale bar: 10 μm.

Noteworthy is that all CRet+ cell types (particularly classes 1, 2, 3, 5, and 8) had dendrites closely associated with blood vessels penetrating the pia (Figures 2L,Q, 4M, 5D,E, 6A–C, **11F**, **14G,H**).

### Comparative dendritic morphometry identifies SPF cells as a unique class

The quantitative results indicated significant differences in the dendritic architecture of class 1–8 CRet+ neurons in layer 1 (Figures 3G–I). A salient feature of cells in classes 2–5 were short lengths of lower order (< 4^•^) dendritic segments (Figure 3G), with the vast majority of the corresponding branch points being located proximal to parent somata (Figure 3H). Also evident were the long total lengths of the complex dendritic arbors of class 2 neurons and the relative simplicity of class 7 unipolar cells (Figure 3G).

With regard to class 8 SPF cells, the dendrogram plots illustrate the frequent presence of higher dendritic orders (>7^•^) — a characteristic not found in other classes. Indeed, the mean total length of SPF cell higher order dendrites (>150 μm from soma) was significantly greater than all other cell types, notably class 1/2 neurons [Figure 3G; class 8 > class 2: +24.3% ± 1.82 (mean ± SEM, *n* = 8); *t*-test *p* < 0.01].

Branch point distribution analyses (Figure 3H) showed a significantly greater (p < 0.01) number of dendritic branch points in the periphery of SPF dendritic arbors as a function of maximum dendritic arbor length (100%) compared with other neuron classes. [Mean branch point distribution values (%) were: Class 1, 40.3 ± 5.8 (mean ± SEM; *n* = 12 cells per class); Class 2, 23.1 ± 3.4; Class 3, 25.8 ± 3.9; Class 4, 33.6 ± 5.6; Class 5, 15.4 ± 3.4; Class 6, 29.3 ± 1.6; Class 7, 0; and Class 8, 69.4 ± 4.6].

WeST analyses of CRet+ cell classes in layer 1 were as follows: Class 1, 4.1 ± 0.2 (mean ± SEM; *n* = 10 cells per class); Class 2, 6.4 ± 0.2; Class 3, 3.9 ± 0.1; Class 4, 5.2 ± 0.2; Class 5, 5.7 ± 0.5; Class 6, 2.9 ± 0.3; Class 7, 1.0 ± 0.2; and Class 8, 8.9 ± 0.2. Statistical analyses indicated that the mean WeST index of class 8 SPF cells was significantly higher (*p* < 0.01) than all other cell types (Figure 3I).

In sum, these results highlight SPF cells as a specific morphometric class of CRet+ neuron in layer 1 of adult monkey mPFC. Further, as indicated in the orientation plot shown in Figure 3J, the dendritic arbors of SPF cells were markedly polarized about their somata.

### Class 8: “subpial fan (SPF) cells”

#### Light microscopical features of identified SPF cells

##### Areal/sublaminar location and frequency

**Figure 7:**
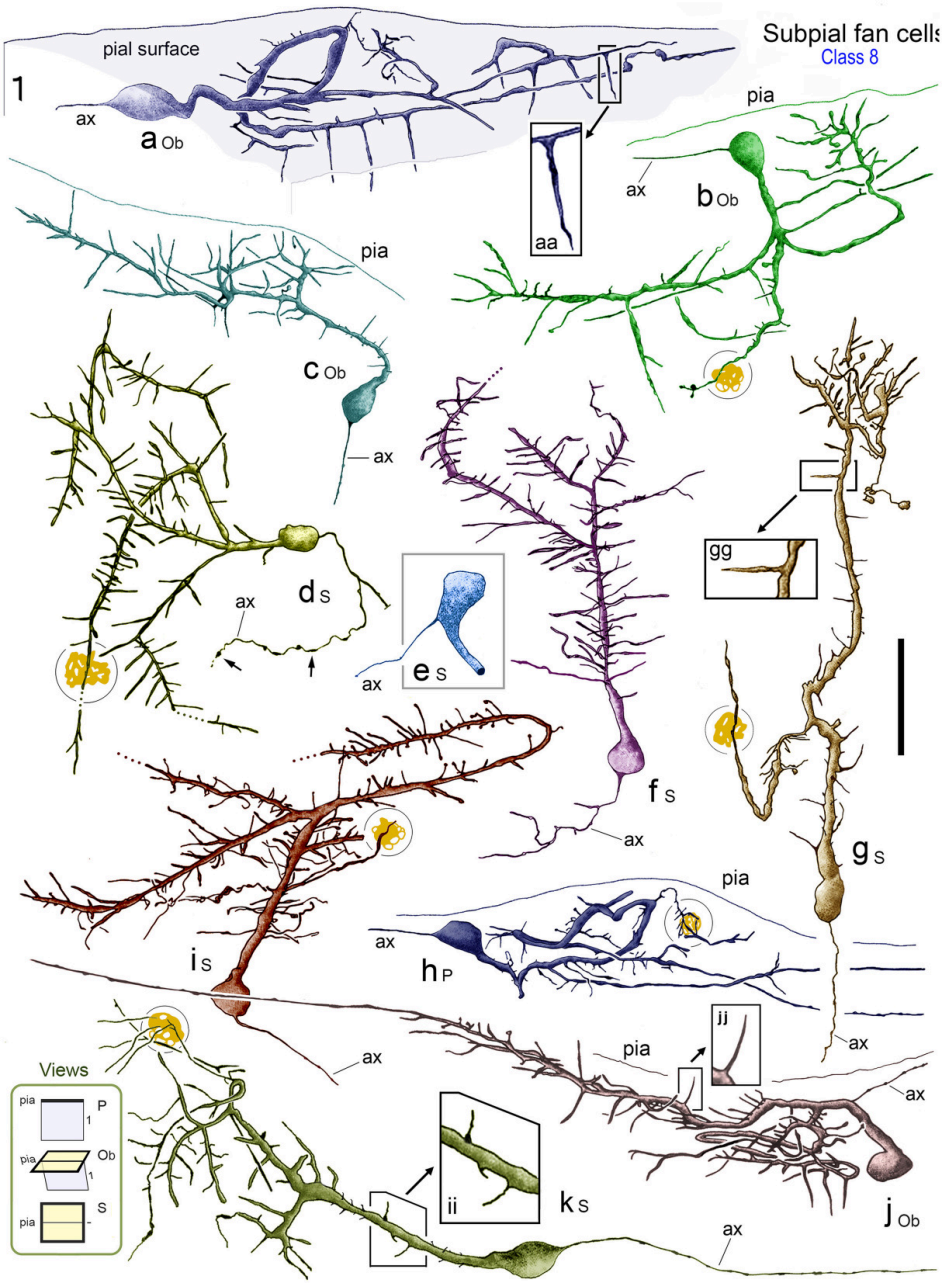
**Reconstructions of class 8 SPF cells (a–k) in adult monkey PFC illustrating the broad range of dendritic morphologies**. Boxed insets show enlarged examples of the thorn-like side branchlets that arise from the main processes of cells a, g, and j. All cells lay immediately below the pial surface and possessed a prominent main dendrite that coursed tangentially within upper layer 1. [Lower left inset codes viewing planes with respect to the plane of the pial surface as: P, perpendicular; Ob, oblique; S, surface]. All cells had a single fine axon-like processes (ax) emerging either from the opposite somatic pole (cells a, c, d, f, g, h, i), from the side of the soma (cell b), base of the main dendrite (cell e) or proximally along the primary process (cell j). Axonal varicosities are indicated (small arrows). Note the long thorns of cells a, g and j and the terminal interwoven dendritic processes of cell g (c.f. cells in Figure 8). Some cells had lipofuscin-rich regions associated with their dendrites (shown encircled). Areal locations of cells: a, area 46d; b, area 25; c, area 32; d, area 24a; e, area 46d; f, area 32; g, area 24c; h, area 32; i, area 46v; j, area 9; k, area 24c. Scale bar: 50 μm.

SPF cells occurred across all PFC areas being more common in the depths of the cingulate and principal sulci (Figure 1B). They were also present in other areas beyond PFC — for example, temporopolar (area 38), anterior insular, and orbitofrontal cortices (Figure 1B). Their number was highly variable across individuals, being more numerous in younger cases (see below). Cells lay immediately beneath the pial surface or in upper layer 1 (Figures 3E,F, 4, 5E, 6A–E, 7, **9A,A′**, **13A,H**, **14F**).

The somata of SPF cells were situated millimeters apart (c.1–10 mm) and could be found clustered with other CRet+ neurons in upper layer 1 (Figure 5I). They occurred with a linear density of approximately 2–5 per 10 mm of pia (estimate derived using 200 μm thick sections).

##### Shape and size of somata

**Figure 8:**
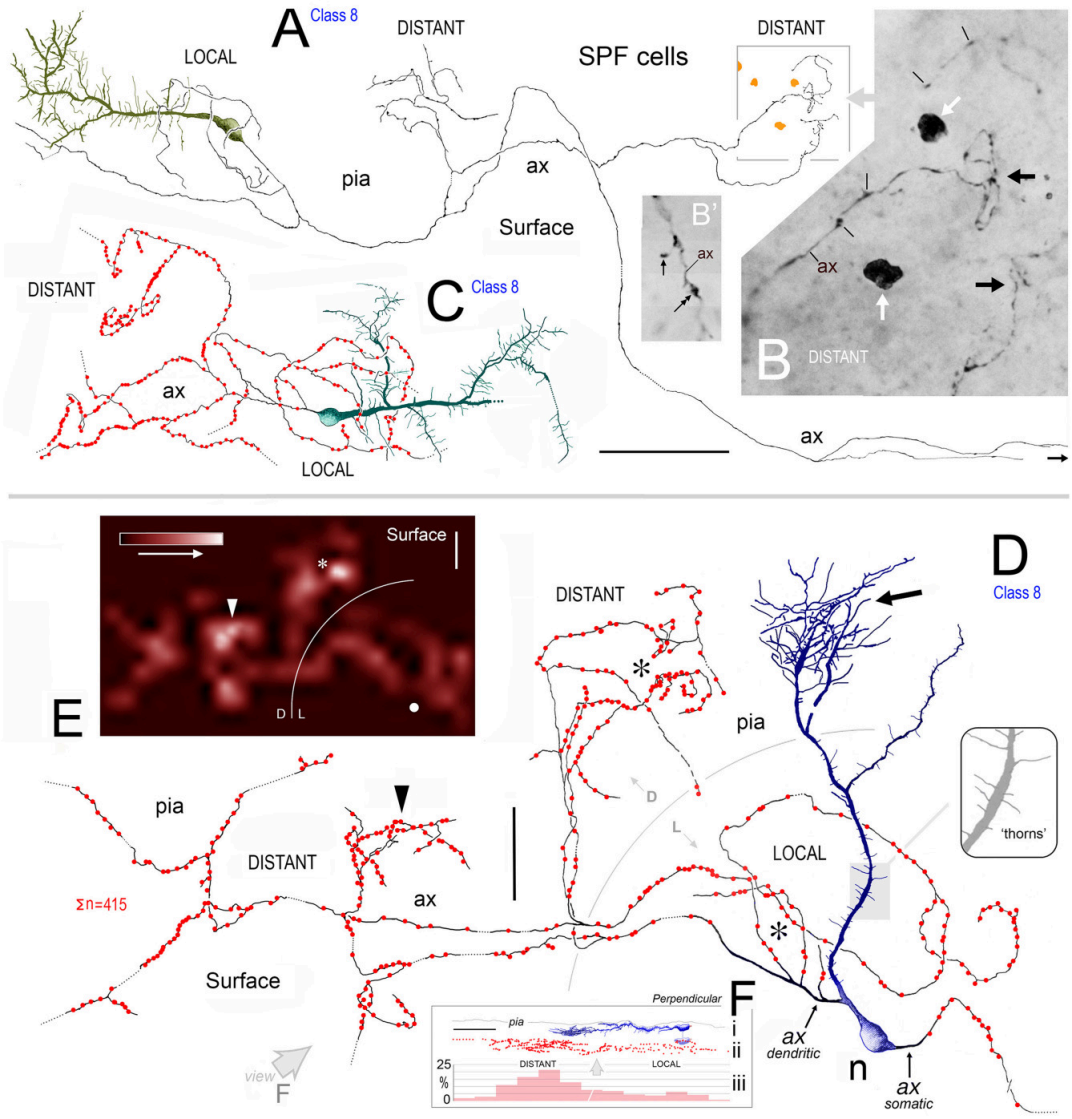
**(A)** View of pial surface in area 32. A class 8 SPF cell gives rise to a tangential axonal plexus that ramifies both proximally (LOCAL) and distally (DISTANT) to the soma. **(B)** Boxed region in **(A)** showing numerous varicosities (arrows) along two distal segments of the axon (ax). Dark irregular profiles of cellular debris (white arrows). **(B**′**)** Axonal boutons — *terminaux* (arrow) and *en passant* (double headed arrow). **(C)** Surface view of a class 8 SPF cell in area 9. Individual axonal swellings along the proximal and distal sections of the axon are indicated (red dots). Note fan-like dendritic trees of both SPF cells. Scale bar for **(A,C)**: 100 μm. **(D)** Tangential view of a class 8 SPF cell (n) in area 24b with a fine varicose axonal process (ax) emerging from one somatic pole (*ax somatic*). The other pole gives rise to a proximally bifurcating thick primary dendrite with numerous dendritic “thorns” (shaded region and inset) and terminates in a fan-like plexus of fine processes (arrow). A proximal dendritic branch bifurcates into several fine varicose axonal fibers (*ax dendritic*) that ramify extensively — both locally and at a distance from the soma. Boundary (150 μm radius from soma) between LOCAL (L) and DISTANT (D) zones is indicated. The morphologies of these processes are similar to those seen in **(A,C)**. Scale bar: 50 μm. **(E)** Density distribution of axonal varicosities from neuron in **(D)**. (Lattice resolution 20 × 20 μm squares). White arrow head indicates cluster of varicosities indicated in **(D)**. Soma (white dot). Scale bar: 50 μm. **(F)** Perpendicular view of thorn cell viewed along axis indicated (open arrow in **D**). (i) dendritic arbor, (ii) distribution of axonal varicosities relative to somatic profile, and (iii) frequency of axonal varicosities within 50 μm wide sampling bins — note distribution in distant and local zones. Scale bar: 100 μm.

**Figure 9:**
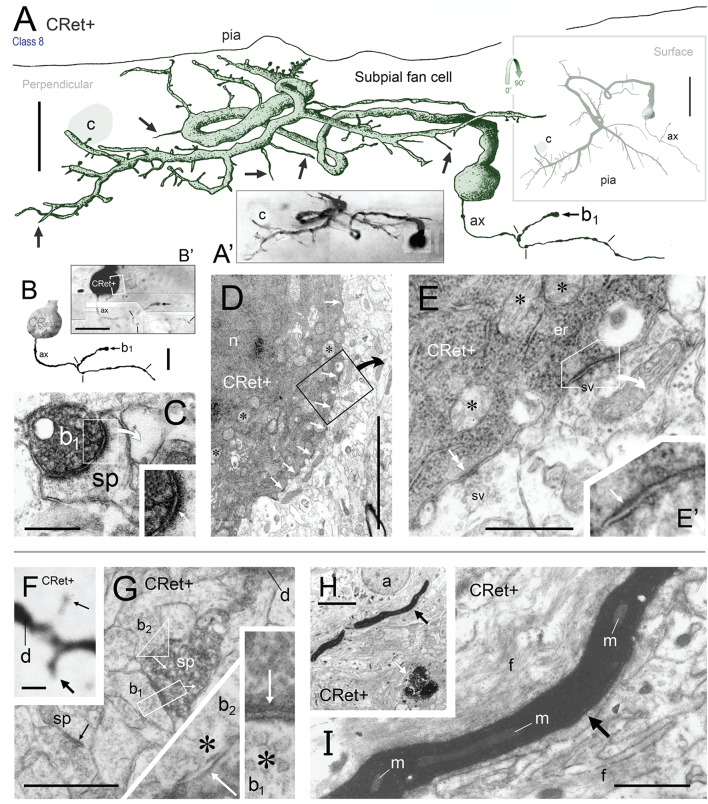
**(A)** Oblique view of a reconstructed class 8 SPF cell in area 46v. Note the unipolar soma and the numerous thorns (arrows) arising from the dendrites. The labeled initial axonal segment (ax) and several varicosities (lines) are indicated. One terminal swelling (b_1_) is highlighted (arrow). The profile of a capillary (c) is shown. Scale bar: 25 μm. **(A**′**)** Photographic montage of the SPF cell in **(A). (B)** Drawing highlighting the axon initial segment of the CRet+ cell in **(A)**. Scale bar: 10 μm. **(B**′**)** Photomicrographic montage of initial portion of axon. Axon bouton b_1_. **(C)** Correlated ultrastructural analysis revealed CRet+ bouton b_1_ formed a synaptic junction with the cupped profile of a dendritic spine head (sp). Scale bar: 1.0 μm. Inset: High magnification image showing the asymmetric (A-type) nature (arrow) of the junction seen in **(C)**. **(D)** Electron micrograph of part of the CRet+ SPF soma seen in **(A,B)**. The soma receives a very high number of closely positioned synaptic contacts from unlabeled boutons (CRet-) (white arrows). Abundant mitochondia (asterisks), endoplasmic and hordes of ribosomes are present in the cell cytoplasm (asterisks). Nucleus, n. The boxed region is shown at higher magnification in **(E)**. Scale bar: 5.0 μm. **(E)** An unlabeled A-type synapse innervates the SPF soma (see **E**′). Elsewhere a CRet- bouton forms a S-type axosomatic junction (double headed arrow). Mitochondria (m) and rough endoplasmic reticulum (er) are indicated. Synaptic vesicles (sv). Scale bar: 1.0 μm. **(E**′**)** High magnification of the A-type axosomatic junction in **(E)** (arrow). **(F)** CRet+ dendrite (d) issuing a large headed dendritic spine (arrow) and a fine spine (thin arrow — away from focal plane). Scale bar: 2.0 μm. **(G)** Correlated ultrastructure of the large spine (sp) in **(F)** receiving A-type input from bouton b_1_ and S-type input from bouton b_2_. Both pre-synaptic boutons are unlabeled. Synaptic vesicles (asterisk). Scale bar: 2.0 μm. **(H)** CRet+ filamentous process (arrow) from a SPF cell. Electron-dense profile of cellular debris (white arrow). Astrocyte, a. Scale bar: 5.0 μm. **(I)** Enlargement of the filament in **(H)**. Note mitochondrial profiles. Numerous unlabeled fine filamentous strands (f). Scale bar: 5.0 μm.

SPF cells possessed smooth somata that were ovoid, pyriform or circular in outline (max/min caliper diameter ratio = 1.2 ± 0.4; mean ± SEM, *n* = 32: see Figures 3–**9**). Somatic sizes ranged from 15.3 to 30.8 μm (d.circ 21.4 ± 2.2 μm; mean ± SEM, *n* = 22).

##### Dendritic architecture

The characteristic feature of these unipolar neurons was a single horizontal primary dendrite that usually gave rise to numerous repeatedly dividing daughter branches with increasingly shorter segment lengths — giving the whole dendritic arbor the appearance of a “corymb-like” fan (Figures 3G, 5A, 7). The main dendrite of some cells extended for over 200 μm before branching into a radiate spray of higher order segments (Figures 4F, 6E, 7 — cell g, 8D).

Dendrites had perpendicular thorn-like branchlets (Figures 3F, 5A,E,F, 6B,E–G; 7 — cells a,g,j,k; 8D, **10A**). These dendritic side-branches (5–25 μm in length) were frequently aligned vertically in layer 1. Their number varied widely across SPF cells and were most common proximally — occurring with a linear density of 0.69 ± 0.55 per 10 μm (mean ± SD). None of these branchlets ended in bulb-like expansions (c.f. Figure 2J).

In addition, lower order dendrites could give rise to numerous fine caliber filamentous strands of varying lengths (Figures 6B,F,G, 7 — cells f,i, 8A). These willowy strands were much thinner and usually longer than the thorns described above (Figures 6F,G). Proximal dendrites also gave rise to low numbers of thin- and mushroom-type dendritic spines (0.24 ± 0.03 spines per μm; mean ± S.D.; Figure 6H). Distal dendrites could narrow gradually into varicose “axon-like” processes (Figures 5E,F, 7 — cell J, 8D).

3D reconstructions/rotations confirmed that the dendritic (and axonal) processes of SPF cells were oriented parallel with the pial surface and lay flat directly beneath the pia (Figures 4M, 5C,H,I, 7, 8, 9A).

##### Axonal architecture

Axons of SPF cells originated from various locations over their somata and proximal dendrites (Figures 3F, 5–7, 8D). The extent of axonal immunolabeling varied between short segments (Figures 5–7) to extensive arbors (Figure 8). In well-labeled SPF cells, local axons were mainly distributed in the vicinity of parent somata with more distal segments forming interwoven plexi (Figure 8). Morphometric data indicated that the tangential areal density of axonal swellings per 2500 μm^2^ of cortical surface was significantly higher (+57%; p ≪ 0.01) in distant zones (20.7 ± 1.2; mean ± SEM, *n* = 8 cells) than in local zones (13.2 ± 0.8; Figure 8F). Axons possessed boutons *en passant* and *terminaux* (Figure 8B′).

3D reconstructions indicated that SPF cell axonal arbors ramified tangentially in upper layer 1 over wide expanses of territory (~100,000 μm^2^; Figures 8A,C,D).

The immunolabeled dendritic and axonal arbors of neighboring SPF cells did not overlap.

##### SPF cells in close association with capillaries

A conspicuous feature of SPF somata and dendrites was their frequent close association with blood vessels penetrating layer 1 from the pial surface (Figures 5D,E, 6A–C, 9A, **11F**, **13F**, **14F–H**).

##### CRet+ punctae associated with SPF cells

CRet+ punctae (presumed pre-synaptic boutons) of unknown origin were found in close contact with the dendritic arbors of SPF cells (Figures 5E,H,G,G′,I). Three SPF cells had a single CRet+ axonal swelling closely opposed to their somata (see below — **Figures 11F,G**).

#### Ultrastructure of identified SPF cells

##### Somata

Cell bodies possessed a single nucleolus within a heavily infolded large nucleus that occupied a large proportion of the soma (**Figure 11F**). The cytoplasm contained a typical array of subcellular organelles (Figures 9D,E; see Peters et al., 1991). A high number of axosomatic inputs were present comprised of both asymmetric (A-type; 21% occurence) and symmetric (S-type; 79%) junctions (Figures 9D,E). S-type input came from CRet- presynaptic boutons whereas A-type input was derived from mainly CRet- and occasional CRet+ boutons (Figures 9D,E, **11F,G**).

##### Dendritic shafts

Labeled dendritic shafts contained microtubules and mitochondrial profiles (**Figure 11K**). Unlabeled A-type and S-type axodendritic synapses were present — however, their distribution was uneven: A-type junctions were more frequent (1.3x) distally whereas S-type junctions were more common (1.5x) proximally, especially over primary dendritic shafts. S-type junctions were formed by both CRet+ and CRet- boutons formed S-type junctions.

##### Spines

The profiles of dendritic spines varied in shape and size (stem lengths/head diameters) — ranging from stubby protrusions, to “lollipop” and long thin drumstick profiles up to 3 μm in length. All spines investigated (*n* = 21) received CRet- A-type synaptic input with 71% (*n* = 16) found to receive additional CRet- S-type input (Figures 9F,G).

##### Filamentous processes

These processes were typically thin (< 3 μm) and extended for considerable distances into the surrounding neuropil (Figure 9H). The profiles of long mitochondria were central components of these structures (Figure 9I).

##### Thorns

**Figure 10:**
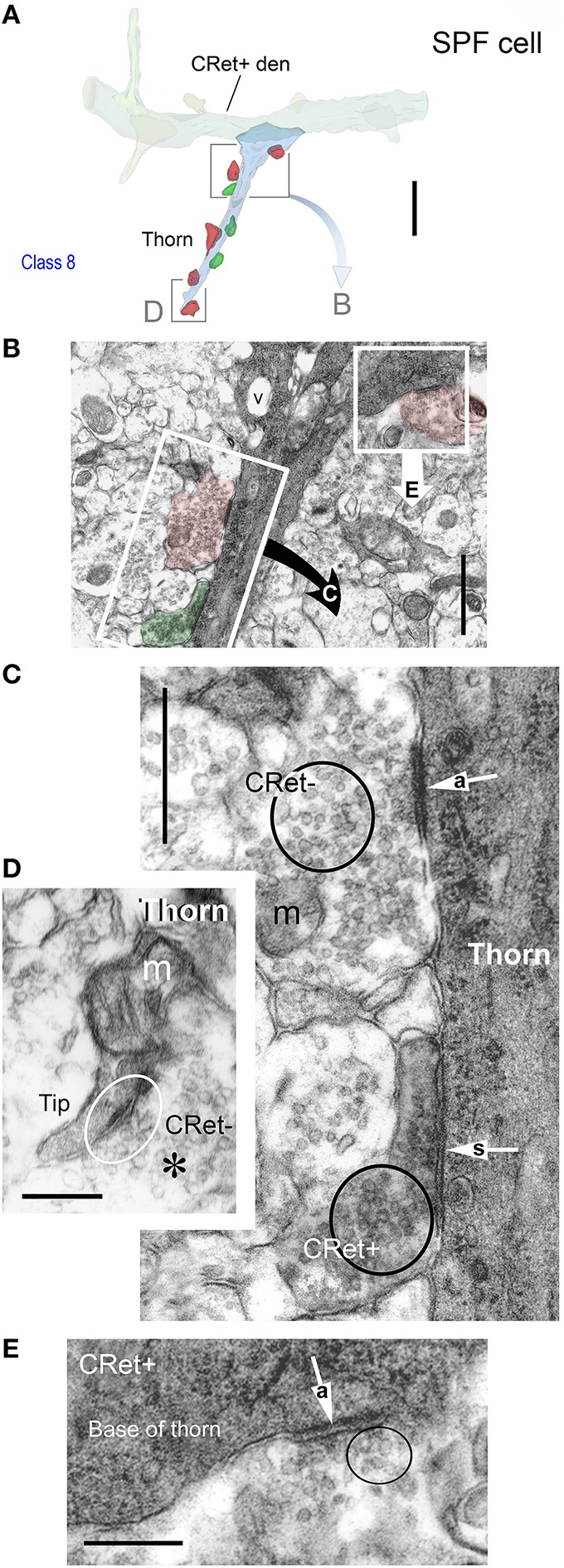
**(A)** 3D ultrastructural reconstruction of a SPF cell dendritic segment with a long thorn-like process (blue) emerging from the shaft. Only pre-synaptic boutons apposing the thorn are indicated: red boutons form A-type synapses, green boutons S-type junctions. The framed areas are shown in **(B,D)**. Scale bar = 5 μm. **(B)** Ultrastructure of the framed area in **(A)**. [The framed regions in this figure are shown in **(C,D)**, respectively]. Note vacuole (v) in thorn base. Scale bar = 1 μm. **(C)** An unlabeled (CRet-) bouton establishes an A-type (“a” white arrow) synaptic junctions with the CRet+ immunolabeled dendritic thorn. A CRet+ labeled bouton make a S-type (“s” white arrow) with the same target. Clusters of synaptic vesicles are indicated in the pre-synaptic elements (black circles). Mitochondrion, m. Scale bar = 0.5 μm. **(D)** Ultrastructural features of the framed area in **(A)**. The tip of the dendritic thorn is filled with a mitochondrion and the narrow distal portion receives an A-type synaptic contact (encircled) from an unlabeled (CRet-) bouton. Synaptic vesicles (asterisk). Scale bar = 0.25 μm. **(E)** An unlabeled bouton located at the base of the dendritic thorn (see **A**) establishes an A-type (“a” white arrow) synaptic junction (encircled) with the CRet+ labeled dendritic process. Scale bar = 0.25 μm.

Dendritic thorns originated as prominences from the profiles of dendritic shafts (Figures 10A,B). The base of thorns contained mitochondria and clusters of ribosomes (Figures 10B,E). These organelles continued into the stems of thorns assuming positions alongside streams of longitudinally running filaments. Mitochondria were present throughout their length (Figure 10D). Thorns received both A-type (65% occurrence) and S-type (35%) synaptic contacts (Figures 10A,C,D). A-type input was from CRet- boutons; and S-type input from both CRet+ and CRet- boutons (Figures 10B,C). CRet- A-type junctions were commonly found either adjacent or opposite the entrance to thorns (Figures 10B,E).

##### Axonal connectivity

**Figure 11:**
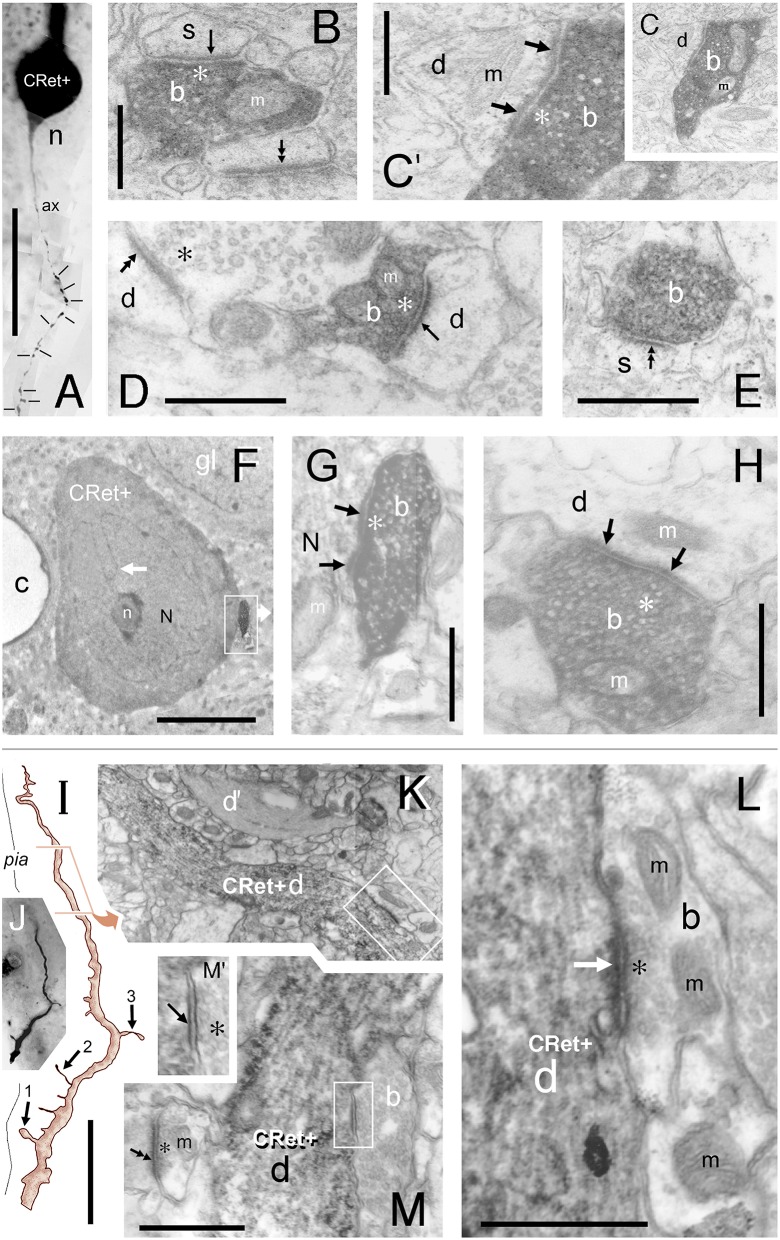
**(A–H)** “*Local” and “distant” synaptic connectivity of identified SPF cell axonal boutons:* **(A)** Area 32: Photographic montage (tangential plane) of a CRet+ SPF cell body (n) and the initial segment of the axon (ax). Note the numerous varicosities (lines) along axonal process. Scale bar: 50 μm. **(B)** CRet+ immunolabeled presynaptic bouton (b) establishes an A-type synapse (arrow) with an unlabeled (CRet-) profile of a spine head (s). Numerous synaptic vesicles (asterisk) and a mitochondrion (m) are present in the presynaptic element. Another A-type axospinous synapse is indicated (double headed arrow). Scale bar: 0.5 μm. **(C)** Immunolabeled presynaptic bouton (b) establishes an A-type synapse (arrow) with the profile of an unlabeled dendritic shaft (d). **(C**′**)** High magnification image showing the asymmetric nature of the synapse between the neural elements seen in **(C)**. Synaptic vesicles in the presynaptic labeled bouton are indicated (asterisk). Mitochondrion (m). Scale bar: 0.5 μm. **(D)** CRet+ bouton (b) from the neuron seen in **(A)** establishes an asymmetric synaptic junction (arrow) with a small unlabeled dendritic shaft profile (d). Another asymmetric axodendritic synapse is indicated (double headed arrow). Mitochondrion (m). Scale bar: 0.5 μm. **(E)** CRet+ bouton **(b)** from neuron in **(A)** forms an A-type synaptic junction with a spine profile (s). Scale bar: 0.5 μm. **(F)** Soma (N) of an CRet+ immunolabeled SPF cell. The soma is innervated by a strongly immunoreactive CRet+ bouton (boxed region, shown enlarged in **G**). Infolded nucleus (white arrow). Nucleolus, n; capillary, c; glial cell, gl. Scale bar: 50 μm. **(G)** The CRet+ bouton seen in **(F)** establishes an A-type synapse (arrow) with the soma of the SPF cell. Synaptic vesicles are indicated (asterisk). Mitochondrion, m. Scale bar: 0.5 μm. **(H)** Labeled CRet+ bouton (b) forms an A-type synaptic junction with an unlabeled dendritic shaft (d). Mitochondria are indicated (m). Scale bar: 0.5 μm. **(I–M)** *Synaptic input to SPF dendrites:* **(I)** Reconstruction of a CRet+ SPF cell dendritic process. Indicated are two spinous protrusions (1 and 3) and a thin drumstick spine (2). The ultrastructure of the outlined segment is shown in **(K)**. Scale bar: 10 μm. **(J)** Photomicrograph of the labeled CRet+ dendrite in **(I). (K)** Electron micrograph of the labeled dendritic segment (CRet+ d) outlined in **(I)**. An unlabeled dendritic profile is indicated **(d**′**)**. The boxed region is shown in **(L)**. Scale bar: 1.0 μm. **(L)** A CRet- bouton (b) establishes an A-type synaptic junction (white arrow) with the labeled dendritic shaft (CRet+ d). Synaptic vesicles in the presynaptic bouton are indicated (asterisk). Mitochondria, m. Scale bar: 1.0 μm. **(M)** A CRet- bouton (b) makes a S-type synaptic junction (boxed region) with the CRet+ labeled dendritic shaft (d). Elsewhere, an unlabeled synaptic bouton makes an A-type synaptic junction (double headed arrow) with a fine caliber dendritic shaft. Mitochondrion, m. Scale bar: 1.0 μm. **(M**′**)** Higher magnification of the synaptic junction boxed in **(M)**. Synaptic vesicles (asterisk).

SPF cell axonal arbors were unmyelinated and *divided* into local and distant segments — with the areal density of axonal varicosities differing significantly between zones (see above). Following correlated light and electron microscopical analyses, identified axonal varicosities along these axons were confirmed as CRet+ synaptic boutons establishing exclusively A-type junctions with CRet- post-synaptic targets (Figures 9B,C, 11A–H). These boutons contained predominantly round synaptic vesicles (Figures 9G, 10C, 11A–H). CRet+ boutons of unknown origin were found throughout layer 1 neuropil (Figure 5G) establishing S-type synaptic contacts with unidentified targets (not illustrated).

Table 1 provides a quantitative analysis of the cellular compartments post-synaptic to CRet+ boutons from identified SPF cells — data are presented separately for “local” and “distant” segments. The post-synaptic targets were mainly dendritic shafts (*sh*) and spines (*sp*) — with the *sh/sp* ratio 2.1 locally and 3.7 distally. Analyses of serial sections indicated that a maximum of 3 axonal boutons were found to innervate a single post-synaptic target and that most (>85%) of the axodendritic synapses innervated *sparsely-spiny* thin-caliber processes (Table 1). Of note, the three axosomatic contacts were onto different somata which received both A- and S-type input.

**Table 1:** Post-synaptic targets of identified layer 1 CRet+ SPF cell axonal boutons^*^.

**Compartment**	Local^**^						Distant^**^					
	**sp^†^**	**sh^†^°**	**cb**	**ais**	**?**	**n (∑)**	**sp^†^**	**sh^†^°**	**cb^^^**	**ais**	**?**	**n (∑)**
Abs. N°	22	47	0	0	5	74	15	56	3	0	5	79
%	29.7	63.5	0.0	0.0	6.8	(100)	19.0	70.9	3.8	0.0	6.3	(100)

* Ultrastructural data derived from 74 “local” and 79 “distant” axonal boutons sampled from five identified CRet+ SPF cells in four animals. Post-synaptic compartments: sp, dendritic spine; sh, dendritic shaft; cb, cell body; ais, axon initial segment; ?, uncertain profile identity (dendritic spine v fine-caliber dendritic shaft). All boutons formed A-type synaptic junctions.

** All post-synaptic targets were CRet immunonegative (CRet-).

† “sh/sp” ratio: Local = 2.14; Distal = 3.73.

° Many of the axodendritic contacts (91/103 = 88%) were onto fine-caliber sparsely spine-bearing dendritic shafts; 12% onto smooth dendritic shafts.

^ Three separate cell bodies innervated. All somata received A- and S-type inputs.

**Figure 12:**
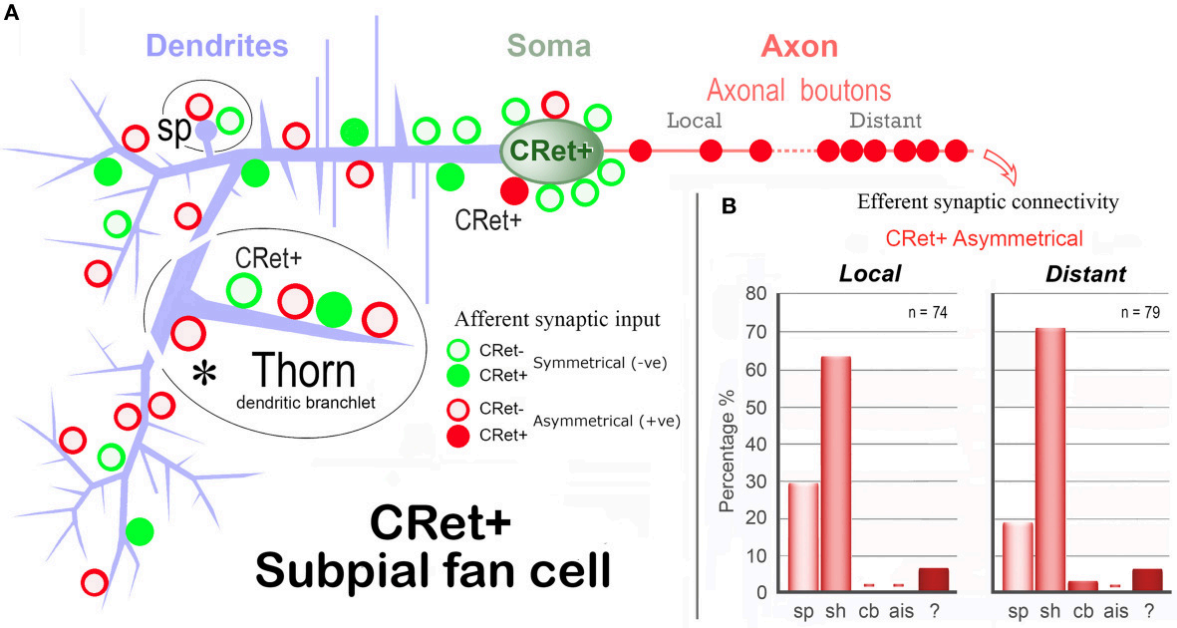
**Summary diagram of the afferent and efferent synaptic connectivity of identified SPF cells in adult monkey PFC. (A)** *Dendrites:* Asymmetric (A-type) synaptic inputs from CRet- boutons are more frequent along distal dendritic shafts, with symmetric (S-type) synapses more common on proximal shafts. (S-types inputs are derived from both CRet+ and CRet- boutons). Dendritic “thorns” are richly innervated by A-type (CRet-) and S-type (CRet- and CRet+) synaptic inputs. The bases of thorns receive CRet- A-type input (asterisk). Dendritic spines (sp) receive A-type and S-type (both CRet-) inputs. *Soma:* Synaptic input to the soma derives from A-type and S-type boutons. A-type axosomatic boutons are subdivided into CRet+ and CRet- types — with the latter predominant. (CRet+ A-type boutons were not derived from SPF cells). *Axonal boutons:* CRet+ boutons establish exclusively A-type junctions with CRet- post-synaptic targets. **(B)** The summary bar charts show the post-synaptic compartments innervated by SPF cell axonal boutons (Table 1). Data for axonal boutons lying “local” and “distant” to parent somata are given separately. Post-synaptic compartments: dendritic spine, sp; dendritic shaft, sh; cell body, cb; axon initial segment, ais; and unidentified elements?

A summary map of the synaptic connectivity of SPF cells is presented in Figure 12.

### Cellular debris and degenerating SPF cells in layer 1

**Figure 13:**
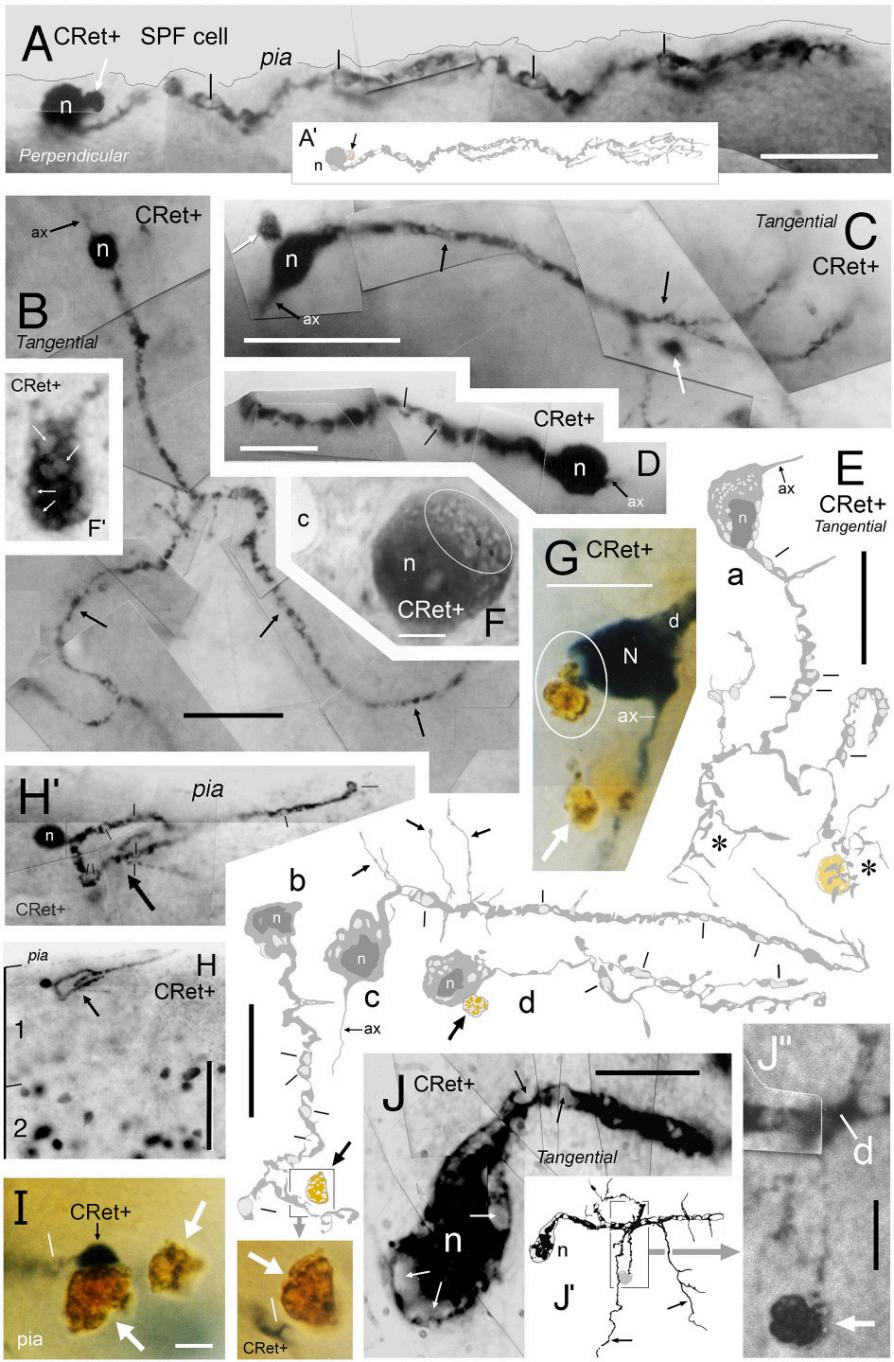
***Degenerating class 8 — SPF cells*. (A)** Perpendicular view of a degenerating SPF cell in area 46v. (Reconstruction of cell in **A**′). Vacuoles in the main dendritic processes (lines). Note the dark profile (white arrow) abutting the soma (n). Scale bar: 25 μm. **(B–D)** Surface (tangential) views of three unipolar CRet+ SPF cells with vacuolated primary and secondary processes (arrows). Presumptive axon-like processes (ax) emerge from opposite poles of the labeled somata in **(B,C)**. (**B**, area 9; **C**, area 25; **D**, area 24b). In **(C)**, dark circular profiles are indicated (arrows): Scale bars: **(B,C)** 50 μm, **(D)** 25 μm. **(E)** Surface (tangential) views. Drawings of CRet+ unipolar thorn cells with vacuolated processes. Several vacuoles are indicated (lines). Note the vacuolated remnants of two regions (asterisks) of terminal processes in cell a (compare with cell n_2_ in Figure 6, cell g in Figure 7, and the cells in Figures 8A,C). Positions of dark lipofuscin profiles (thick arrows) indicated (thick arrows — see also photographic insert) Cell nuclei (n). (Areal locations of cells: a, area 32; b, area 24a; c, area 46d; d, area 9). Scale bars: 25 μm. **(F,F**′**)** Area 32. Degenerating CRet+ somata lying beneath the pia. Numerous discrete ovoid regions in the cytoplasm of both cells are devoid of immunoreactivity (thin white arrows). Capillary, c. Scale bar: 5 μm. **(G)** Yellow/brown lipofuscin-rich profile abutting the soma of a degenerating neuron (outlined). Note disruption of the SPF cell body. Dendrite, d; axon, ax. [CRet+ immunoreactivity visualized using gray (Vector SG) peroxidase substrate]. Scale bar: 20 μm. **(H)** Area 46d. Degenerating CRet+ SPF cell (arrow). Numerous CRet+ neurons are present in layer 2. Scale bar: 100 μm. **(H**′**)** Higher magnification image of the neuron indicated in **(H)**. The primary dendrite courses toward the viewer and is obscured by the vacuolated secondary and higher order dendrites (lines). **(I)** Yellow/brown lipofuscin-rich profiles (white arrows) one of which is in close contact with a degenerating dendrite. (Vacuole, line). Scale bar: 5 μm. **(J)** Area 32. Photographic montage of a degenerating SPF cell body. Numerous vacuoles are present in the soma (white arrows) and in proximal portion of the main process (lines). Scale bar: 10 μm. **(J**′**)** Drawing of the neuron seen in **(J)**. Fine perpendicularly disposed processes arising from the main vacuolated dendrite (arrows). **(J**″**)** Photomicrograph of the region boxed in **(J**′**)**. A fine caliber process derived from the main dendrite (d) comes into close association with a dense cellular profile (arrow). Scale bar: 5 μm.

**Figure 14:**
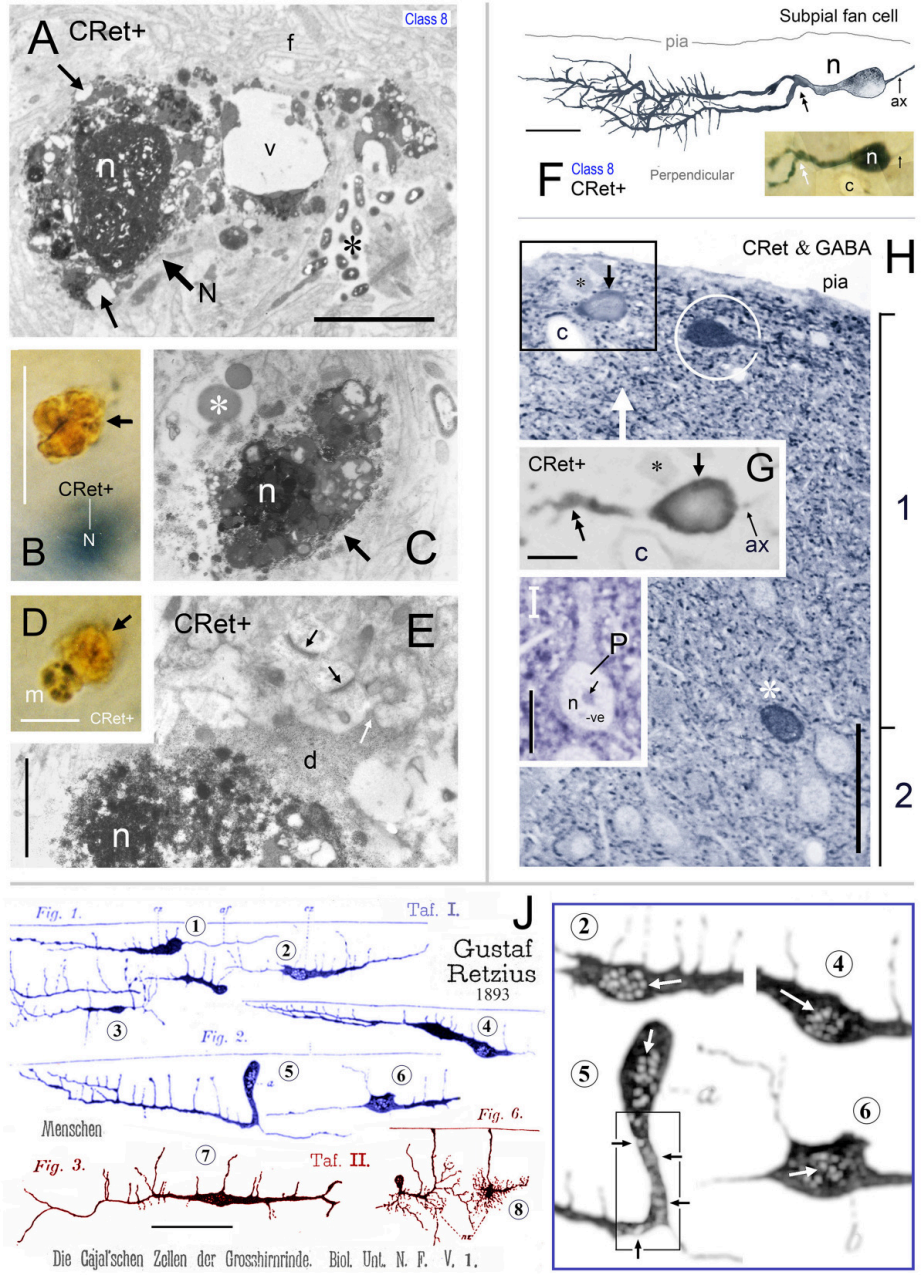
**(A)** Area 24b. Somatic profile of an identified CRet+ SPF cell (N) in layer 1. Lipofuscin granules and vacuoles in the cytoplasm are indicated (black arrows). Nucleus, n. Large vacuole (v). Fine filamentous astroglial processes in layer 1 (f). Dense membranous inclusions in a dendrite are visible (asterisk). Scale bar: 5 μm. **(B)** Yellow/brown profile of lipofuscin-rich cellular debris (arrow). Note the gray (SG) soma of a CRet+ neuron (N) beneath focal plane. Scale bar: 10 μm. **(C)** Ultrastructural profile of electron-dense cellular profile with a highly condensed pyknotic nucleus (n). Note vacuolated appearance. Large lysosome (asterisk). Scale bar: 5 μm. **(D)** Microglial cell (m — with dark clumped inclusions) associated with lipofuscin-rich cellular debris (arrow). Scale bar: 10 μm. **(E)** Pyknotic neuronal profile (n) with dark cytoplasm (d) that extends into cellular processes (white arrow). Note also normal ultrastructure of asymmetric synaptic junctions in the neuropil (dark arrows). Scale bar: 2 μm. **(F)** Perpendicular reconstruction of a SPF cell (n) in area 24b (perpendicular view). Primary dendritic branch point is indicated (double headed arrow). (Axon, ax). Scale bar: 20 μm. Inset shows SPF cell and a neighboring capillary (c). **(G)** Untreated semithin resin section (2 μm thick) through the somata of the SPF cell in **(F)** (arrow). Positions of fiducial capillary (c) and an unlabeled cellular profile (asterisk) are indicated. The nucleus (n) contains weak immunolabeling. Bifurcation of the primary dendrite is indicated (double headed arrow). Axon, ax. Scale bar: 10 μm. **(H)** Semithin section in **(G)** reacted using post-embedding GABA immunocytochemistry (CRet & GABA). GABA+ cells (white asterisk) have intensely immunoreactive nuclei. Especially note GABA+ fusiform cell beneath pial surface (encircled). Boxed region identifies the CRet+ SPF cell seen in G to be GABA- (i.e., lack of intense GABA nuclear immunolabeling). Axon, ax. (Fiducial markers with **(F,G)**: capillary, c; unlabeled cellular profile, black asterisk). Scale bar: 50 μm. **(I)** Profile of a GABA- pyramidal cell (P) devoid of cytoplasmic and nuclear (n) immunolabeling. Nucleolus (arrow). Scale bar: 10 μm. **(J)**. Drawings by Gustaf Retzius (1893: Plates I and II) showing Golgi-impregnated cells in developing layer 1 of the human neocortex. Retzius illustrates several unipolar cells (e.g., 1, 2, 4, and 5). The somata of cells 2, 4, 5, and 6 are drawn with numerous small ovoid pale regions in the cytoplasm/nucleus (?) — shown enlarged with high contrast in the boxed region (right). These features are similar to degenerating CRet+ cell bodies (see Figures 13E and especially F/F′). Cell 7 is similar to the class 1 cell in Figures 2A–F (this study). Cell 8 resembles the class 4 cell shown in Figure 2O. Scale bar = 100 μm. (The original drawings by Retzius have been altered to display only cells of interest).

In the light microscope, irregular profiles (d.circ. c.5–17 μm) with a dark yellow/orange color (indicative of lipofuscin) were frequently found in the upper tier of layer 1, particularly beneath the pia (Figures 2–8, 13). Such profiles were present in all PFC areas studied and occurred, either singly or in clusters, with a linear density of 1–6 per mm length of pia (Figures 4A,B,D,J,K). They were commonly associated with the processes and somata of SPF cells — as well class 1/2 neurons (Figures 2A,C,C′, 2H–J, 4A,B,I–K, 6A–F, 7, 8B, 13A,C,E, especially **G,I,J**″). Some of the profiles were clearly vacuolated with closely apposed microglial cells (Figures 5E′, 14D). The coloration of these lipofuscin-rich profiles darkened following treatment with osmium tetroxide. Figures 13G,I, 14B show the distinction between gray immunolabeling for CRet alone (using Vector SG kit) and the lipofuscin containing profiles. Accordingly, it was possible to identify a large number of CRet+ SG labeled cellular profiles in upper layer 1 with lipofuscin in their cytoplasmata (Figure 4I).

Interspersed among healthy CRet+ neurons in upper layer 1 were CRet+ cells with somatodendritic compartments at various stages of cytoplasmic vacuolation. Such neurons were most frequently located directly underneath the pia and represented less than 1% of the healthy CRet+ cell population in layer 1. These degenerating neurons were spaced apart with a linear density of 2–10 per 10 mm of pia and were composed of putative Cajal-Retzius cells (Figure 2G) and identified SPF cells (Figures 13A–E,H,H′,J). Noteworthy were subpial CRet+ somatic profiles with unlabeled regions of cytoplasm as illustrated in Figures 13F,F′.

Identified SPF cells undergoing vacuolation were present in all areas examined (Figure 13). The degree of cellular vacuolation varied considerably between SPF cells within an area. Some cells had vacuoles present only in higher order dendrites, others throughout their dendritic arbors, whilst additional cells were almost entirely vacuolated (Figure 13). Where present, dendritic thorns were much swollen (Figure 13E). Heavily vacuolated somata either lacked an axon or possessed the remnants of a dilated initial segment (Figures 13E — cells b,d, G,J). This variation represented a continuum — with approximately five times the number of SPF cells undergoing degeneration in adult cases than those with clear structural integrity (see below).

Ultrastructural analysis of identified vacuolated SPF neurons confirmed that their somata and dendrites were undergoing various stages of structural fragmentation characteristic of cellular degeneration (Figures 14A,E). These somata would contain increased numbers of much enlarged lysosomes, bulky lipofuscin granules associated with regions of vacuolated cytoplasm, dense membranous inclusions and pyknotic nuclei (Figures 14A,E). In the electron microscope, the dark yellow irregular lipofuscin-rich profiles were found to be regions of neuropil with large amounts of condensed cellular debris undergoing advanced stages of degeneration — sometimes containing highly condensed pyknotic nuclei (Figure 14C). Degenerating SPF cells and the clumps of cellular debris in upper layer 1 were surrounded by neuropil with normal structural integrity — for example, containing undisrupted synaptic junctions (Figure 14E).

### Comparison between young adult and adult cases

A preliminary analysis indicated that SPF cells (with normal structural integrity) were more common (approximately x1.5) in younger animals (4–8 years) than older animals (9–12 years). Older adults had more pyknotic neurons (including degenerating SPF cells: Figure 13) and cellular debris in upper layer 1 across all areas.

### Combined pre-embedding CRet and post-embedding GABA immunocytochemistry

Using this procedure the nuclear profiles of all seven SPF cells tested were found to be devoid of specific GABA immunoreactivity — similar to pyramidal shaped profiles in layers 2–6 (Figures 14F–I). After GABA immunolabeling, some neighboring CRet- somatic/nuclear profiles were found to be strongly GABA+; for example, ovoid CRet-/GABA+ cell profiles oriented tangentially in upper layer 1 (Figure 14H).

Quantitative analysis of GABA immunoreacted perpendicular semithin sections from areas 32, 24, and 46v (Figures 1A–E) indicated the peak in GABA- neurons occurred 10–40 μm beneath the pia and at the layer 1/2 border (140–160 μm depth) whereas the peak in GABA+ neurons occurred mid-way through layer 1 approximately 80 μm below pia (Figure 1F).

The ratio of “CRet+/GABA- neurons” (*n* = 17) to “CRet+/GABA+ neurons” (*n* = 83) in layer 1 of area 32 was 0.21 (1:5) (17% of CRet+ neurons were GABA-; Figures 14F–H).

## Discussion

This neurocytological study illustrates the morphological variety of calretinin immunopositive (CRet+) cells in layer 1 of the young adult and adult macaque monkey PFC. The diverse morphologies of these neurons have not been fully classified previously — although the studies of Condé et al. (1994) and Gabbott and Bacon (1996a) document several of the CRet+ subtypes present in the current classification. The survey provided here served as a necessary comparative background for a comprehensive account of SPF cells — the main focus of the study.

### Technical considerations

The investigation used perfusion-fixed adult monkey material with thick sections (upto 250 μm) being prepared in the major sectioning orientations — especially in the plane tangential to the pial surface. Thick sections gave the possibility of unequivocally tracing the intricate dendritic arbors of SPF cells from their unipolar parent somata. These factors coupled with excellent penetration of the CRet antiserum (and other immunoreagents) into tissue immediately subjacent to the pia gave detail-rich immunomorphology in both the light and electron microscopes.

Whilst the dendrites of CRet+ SPF cells displayed strong immunoreactivity their axonal arbors showed marked variation in labeling — ranging from short initial segments (Figure 5G) to darkly labeled expansive arbors with numerous varicosities (Figures 8A,B). The gradation may reflect variations in CRet concentration — sometimes below the level of immunocytochemical detection. Therefore, it is unlikely that the full extents of the immunolabeled SPF cell axonal arbors were consistently revealed.

### CRet+ neuron classes in layer 1

On the basis of light microscopical and morphometric features eight classes of CRet+ neurons in layer 1 of adult monkey PFC were identified. As discussed below, classes 1 and 2 are probably “classical” Cajal-Retzius cells — with the possibility that some *superficial* unipolar class 7 neurons also belong to this group. Class 8 SPF cells may share a common lineage with these neurons. Moreover, evidence (from across mammalian species) indicates that neurons in classes 1, 2, 7, and 8 are probably excitatory in function (Del Río et al., 1995; this study), whereas most neurons in classes 3–6 are likely GABA-containing inhibitory LCNs (Gabbott and Bacon, 1996a,b and unpublished observations; Condé et al., 1994; Varga et al., 2015). Furthermore, class 4 cells are similar to the Golgi-impregnated neurons in human layer 1 described as *neurogliaform cells* by Retzius (1893; see cell 8 in Figure 14J of this study) and as *dwarf stellate cells* by Ramón y Cajal (1899b).

### Class 8 SPF cells in adult monkey PFC

SPF cells represent a morphologically distinct class of CRet+ neuron in layer 1 of adult monkey PFC — with similar neurons present in other areas outside PFC. As far as the author is aware, such cells have not been previously recognized. Golgi-impregnation investigations and other immunocytological studies, using antisera against calretinin, as well as the calcium binding proteins calbindin and parvalbumin, charting the pre- and postnatal cellular development of monkey cortex do not specifically identify neurons with the morphology of SPF cells (Huntley and Jones, 1990; Yan et al., 1995a,b; Gabbott and Bacon, 1996a; DeFelipe, 1997; Del Rio and DeFelipe, 1997; Meskenaite, 1997; Ding et al., 2000). An exception is the study of Condé et al. which illustrates a CRet+ cell in layer 1 of adult monkey PFC with the structural rudiments of a SPF cell and remark on the “*long spine-like processes”* arsing from dendrites — no additional comments are provided (Condé et al., 1994 — Figure 5D).

The reasons for the previous lack of identification are unclear. They may relate to the extreme outer position of SPF cells, their comparative rarity, histochemical staining/immunolabeling procedures, species differences, section thickness and orientation effects. The last two factors would limit the extent of defining morphological features present in a single section — e.g., Condé et al. (1994) used 40 μm cryostat sections. This could lead to SPF cells being considered as simple unipolar neurons (see Figure 5I). Alternatively, if only segments of their dendritic fans are present in thin sections, such fragments could be attributed to the elaborate dendritic/axonal arbors possessed by some Cajal-Retzius cells. This highlights the continuing debate about which layer 1 cells qualify for membership of the special Cajal-Retzius family across development and into adulthood (Meyer et al., 1999; Zečević and Rakic, 2001; Martinez-Cerdeno and Noctor, 2014; Marín-Padilla, 2015: see especially DeFelipe et al., 2013).

Interestingly, Anstötz et al. (2014) and Ma et al. (2014) have studied the electrophysiological properties, neurochemical content and connectivity of Cajal-Retzius cells in the developing cortex of mice. They illustrate the dendritic and axonal arbors of typical and atypical unipolar Cajal-Retzius cells which bear some resemblance to the SPF cells described in adult monkey. However, the somatodendritic compartments of the cells in postnatal mouse reside mostly in deep layer 1 with their extensive axonal arbors ramifying throughout the lamina — unlike SPF cells in monkey.

Are SPF cells present in human PFC? The study of Gabbott et al. (1997) provided evidence of CRet+ Cajal-Retzius cells in layer 1 of aged human mPFC (>60 years). However, CRet+ neurons with the morphology of SPF cells in adult monkey were not observed in the human material examined. The comparatively much older ages of the human cases studied (Amlien et al., 2016), together with post-mortem interval (< 18 h) and immersion fixation effects may underlie the absence of SPF cells in human mPFC.

### The “Cajal-Retzius” family — SPF cells as relatives?

In Golgi-impregnation studies, Ramón y Cajal (1890) describes “*very singular cells”* in cortical layer 1 of neonatal rodents, rabbits and cats. Retzius (1893, 1894) described more elaborate counterparts (sometimes exclusive variants) of the “*Cajal”sche Zellen'* in human primate fetal cortex (Figure 14J) — with Ramón y Cajal (1909) subsequently confirming their presence at postnatal ages. The cells of Retzius and of Ramón y Cajal fall into an expansive polymorphic family; both at different developmental stages and across species. Ramón y Cajal writes: “*[They] show many variations in the shape, number, and direction of the processes”* and “*…there does not exist any rigorous frontier”* (DeFelipe and Jones, 1988). Despite this, two of the main varieties are firmly represented in the present CRet immunocytochemical study of adult monkey PFC (Marín-Padilla, 1984; DeFelipe and Jones, 1988, pp 201–210).

Ramón y Cajal (1890) defines fusiform cells in the upper part of the molecular layer in rabbit cortex: “*The dominant form is that of a perfect spindle, with two long dendrites directed parallel to the fibrillary layer …the rectilinear dendrites give off, almost at a right angle, small ascending branches…The axon is at least double …commonly [emerging] at the point at which the polar dendrites turn to ascend. The axons run in opposite directions …giving off at intervals fine, ascending, branched filaments that come to complicate notably, the plexus of the superficial [sublayer]*” (DeFelipe and Jones, 1988, pp11–12). An example of this cell type in humans drawn by Retzius (1893) is presented here in Figure 14J — cell 7 (c.f. Figures 2A–F this study).

Horizontal cells in human fetal brain are described by Ramón y Cajal as appearing “…*throughout the thickness of the molecular layer in the form of fusiform, triangular, or stellate cells with very long horizontal processes from which, at right angles, emerge an infinity of small ascending branches; [these] end under the pia in a thick varicosity. The more superficially placed cells (marginal cells) are conical, have an external base adherent to the pia and a central pedicle from which emerge very numerous and enormously long processes …it is impossible to distinguish among the various elongated processes any with the characteristics of axons or the [typical] properties of dendrites*” (DeFelipe and Jones, 1988, p153). For examples of this neuronal phenotype in humans see the drawings of Retzius (1893, Figures 2/3 in Plate III and Ramón y Cajal, 1909, Figures 340 and 341)^2^.

The CRet+ cells in classes 1 and 2 conform to the above descriptions of fusiform and horizontal cells, respectively — with the versions in adult monkey being significantly more complex than those in lower mammals.

In addition, *vertically aligned* marginal/pyriform Cajal-Retzius cells are also present during corticogenesis in humans (Ramón y Cajal, 1909 — Figure 341, cell A; Retzius, 1893 — Figures 1/4 in Plate III; Meyer and González-Hernández, 1993 — Figures 5, 6) — however, similar cells were not seen in the non-human primate material investigated here. Further, the studies of Retzius and Ramón y Cajal identify unipolar cells in upper layer 1 of human cortex [Retzius (1893), reproduced in Figure 14J — cells 1, 4 and 5; Ramón y Cajal (1909) Chap. XXIV Figure 338, cell E]. Ramón y Cajal (1909) also describes “*unipolar fusiform cells”* in lower layer 1 of prenatal and newborn mammals (rat, cat, dog). These unipolar neurons have dendrites which branch infrequently and taper gradually along their course — some processes giving out numerous side-shoots with bulbous endings (termed “*thick nodules”* by Retzius) abutting the pia — similar to Figure 2J in this study. Of note, Ramón y Cajal (1899b) identifies a “*rudimentary cell with a short axon”* in layer 1 of the precentral gyrus of a 1 month old child — not dissimilar to the initial portion of cell j in Figure 7 (this study). However, Ramón y Cajal's illustration shows a unipolar neuron with a simple horizontal dendrite from which emerges a short axon (Ramón y Cajal, 1899b — Figure 93, cell E).

Consequently, the superficial horizontal SPF cells identified immunomorphologically in this study of adult monkey, with axons and characteristically branching fan-like dendritic arbors disposed tangentially in upper layer 1, are qualitatively and quantitatively distinct from the Golgi-impregnated *fusiform, horizontal*, and *vertical marginal cells* described by Ramón y Cajal and Retzius (and others) in a variety of species including primates (Marín-Padilla, 1984, 2015; Meyer and González-Hernández, 1993). However, the possibility exists that some of the superficial CRet+ unipolar (Class 7) cells seen here (Figures 2N, 5I) and the unipolar cells illustrated by Retzius (1893; see Figure 14J — cell 5 in this study) and Ramón y Cajal are highly truncated versions of SPF cells.

The likely excitatory function of SPF cells (demonstrated by the lack of GABA immunoreactivity together with specific ultrastructural features such as spines and thorny dendritic branchlets, as well as significant S-type input proximal to the soma and A-type axonal synaptic junctions) aligns them with the special cells of Retzius and Ramón y Cajal and segregates them from GABAergic LCNs in layer 1. Accordingly, SPF cells may be “atypical” members of the persisting CRet+ Cajal-Retzius family — defining a way-point across a broad structural (and probable functional) spectrum in adult monkeys (Džaja et al., 2014; Gil et al., 2014).

### Demise of SPF cells

A limitation of this study is the lack of a full pre-/postnatal timeline for the observations reported here. Nonetheless, clear evidence is presented across all PFC areas studied of degenerating and highly pyknotic SPF cells (Figures 13, 14A,C,E) and neurons of the classical Cajal-Retzius type in young adult and adult monkey (Figure 2G). Indeed degenerating SPF cells were more numerous in the older animals. Interestingly, Retzius (1893) illustrates vacuolated *Cajal'sche Zellen* in human fetal cortex likely undergoing the early stages of cellular degeneration similar to monkey SPF cells (Figure 14J vs. Figure 13E). Peters and colleagues (Peters et al., 2001; Peters and Sethares, 2002) studied age-related structural changes in neurons and glia throughout layer 1 in areas 46 and 17 of adult (5–12 years) and aged (25–35 years) monkeys. These authors describe the ultrastructural characteristics of rare pyknotic neurons in layer 1. Degenerating SPF cells have similar features (Figure 14) and may be represented in this population (see also Derer and Derer, 1990).

One striking structural component of upper layer 1 in adult monkey PFC are deposits of lipofuscin-rich cellular debris (Figures 4I–K) — some being closely opposed to the somata of SPF cells or enveloping their dendrites (Figures 5E, 6E,F, 7, 13). The stereological study of O'Kusky and Colonnier (1982) reports a highly significant loss of layer 1 neurons between birth and young adulthood (0–8 years) in area 17 of monkey. Given the late postnatal maturation of PFC, similar alterations would result in large numbers of degenerating neurons contributing to the widespread deposition of cellular debris in upper layer 1.

### A non-synaptic function of SPF cells?

A non-synaptic function of SPF cells may relate to the close structural proximity of their somata and dendrites with blood vessels. If SPF cells contain reelin (similar to CRet+ Cajal-Retzius cells) they are strategically placed to secrete this protein across the blood brain barrier via endothelial cells into penetrating blood vessels (Perez-Costas et al., 2015). This would impact the post-natal maturation of neural circuitry within the perfusion domain of neighboring blood flow (Blinder et al., 2013; Chai et al., 2015).

### Synaptic connectivity of SPF cells in adult monkeys

**Figure 15:**
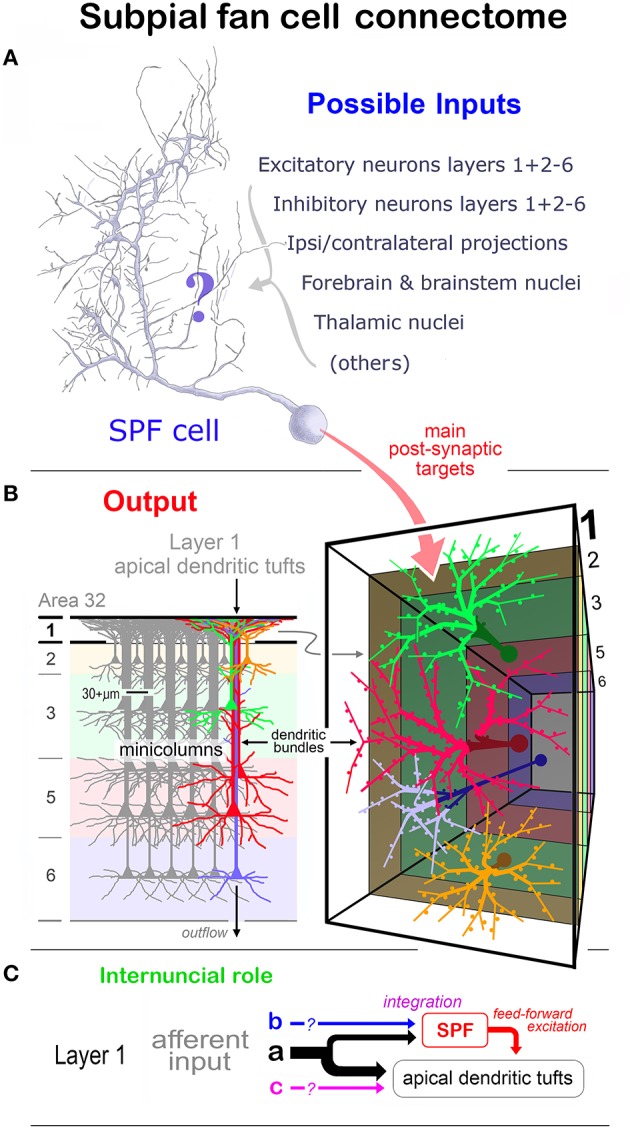
**(A)** Schematic diagram showing possible sources of synaptic input (?) to SPF cells. **(B)** Diagram illustrating the organization of pyramidal cell bodies in agranular area 32 into radial minicolumns (spaced 30+μm apart in adult monkey PFC). The apical dendrites of pyramids in layers 3–6 form bundles as they ascend through the cortex. Layer 2 pyramids contribute to a separate bundling system. Each bundle gives rise to a spray of apical dendritic tufts — the main post-synaptic targets of SPF cells. **(C)** Internuncial role of SPF cells. Extrinsic and intrinsic axonal systems in layer 1 provide common input (a) to the apical dendritic tufts of pyramidal cells and SPF cells. This input may be differentially distributed across cell types. However, SPF cells and dendritic tufts may (?) receive additional input from specific sources (b/c respectively). SPF cells are strategically placed to integrate afferent input and provide a feed-forward excitation influencing neural activity in target apical dendritic tufts.

Figure 12A presents a summary map of synapses over SPF cell dendrites. Although the identities of these inputs are currently undefined, they can be predicted by considering the axonal systems known to ramify in layer 1. A-type inputs to SPF cells are likely derived from (i) other excitatory neurons in layer 1, (ii) the recurrent axonal collaterals of pyramidal cells situated in deeper layers, and (iii) the terminal axonal plexi of long-distance projections from pyramidal neurons in ipsilateral and contralateral cortical areas (Figure 15A; Martin, 1984; Vogt, 2009). In addition, layer 1 in PFC receives direct excitatory drive from specific and non-specific thalamic nuclei which also provide input to mid-layers of the cortex and layer 6 (Jones, 2007). Since no CRet+ boutons with A-type synapses were found to innervate SPF cell dendrites, intralaminar excitatory inputs from other SPF cells and CRet+ Cajal-Retzius cells is unlikely, at least in the adult.

Inhibitory input to SPF cells may come from CRet+ and CRet- GABAergic neurons situated throughout the depth of layer 1 (see Figures 1D,F; also Muralidhar et al., 2014) and from inhibitory LCNs in deeper layers (Figure 15A). For example, evidence in rat and cat when translated to monkey, suggests that S-type axodendritic input to SPF cell dendrites might come from GABAergic double bouquet, bipolar and Martinotti cells in layers 2–6 — these neuron types have ascending axons innervating upper layer 1 (Marín-Padilla, 1971; Ramos-Moreno and Clascá, 2014). Of significance here, Martinotti cells may be either CRet+ or CRet- (Wang et al., 2004). Both CRet+ and CRet- S-type inputs were present over the dendrites of SPF cells with CRet+ S-type boutons located more proximally (Figure 5H). These data suggest the strong proximal influence of GABAergic input on dendritic information processing in SPF cells.

The CRet- S-type synaptic input to SPF cells in layer 1 might also come from cholinergic basal forebrain neurons, and dopaminergic, serotoninergic, histaminergic, and noradrenergic brainstem nuclei (Figure 15A: Mrzliak et al., 1995; Latsari et al., 2002; Janusonis et al., 2004; Jin and Panula, 2005; Huo et al., 2009; Arrovo et al., 2014). Axons from these systems have varicosities that form conventional S-type synaptic contacts as well as numerous swellings without defined specialized membraneous junctions. Of interest are recent studies (Ramos-Moreno and Clascá, 2014; Chen and Kriegstein, 2015) demonstrating long range subcortical inputs to layer 1 originating from GABAergic projection neurons in the zona incerta of rodents having a role in the pre- and post-natal functioning of neurons in the molecular layer.

The dendritic arbors of SPF cells are embedded in the above fiber paths, terminal arborizations and local neural circuits (Figures 15A,C). As mentioned above the pial surface density of SPF cells is likely to be greatly reduced in the adult compared with earlier stages of cortical development — when the processes of functionally active SPF cells (and cells of the Cajal-Retzius family) could intermingle and overlap extensively.

The clustered pattern of boutons along the distal axonal segments of SPF cells (and their prominent innervation of dendritic shafts) may reflect a selective innervation of two apical dendritic systems derived from the known minicolumnar radial organization of the cortex (Figure 15B; Gabbott, 2003). Namely, the distribution mosaic of pyramidal cells in layer 2 (Gabbott et al., unpublished observations; Ichinohe et al., 2003) and the dendritic bundles formed by pyramidal cells in layers 3–6 (Figure 15B, this study; Arikuni et al., 1983; Marín-Padilla, 1990; Schlaug et al., 1995; Gabbott, 2003). Morphometric calculations indicate that about 5000 pyramidal cells distributed in layers 2–6 with apical dendritic tufts that ramify within the small patch of layer 1 innervated by an adult SPF cell axon (~100,000 μm^2^) have a probability (>0) of receiving a synaptic contact (Arikuni et al., 1983; Schlaug et al., 1995; Peters and Sethares, 1996). In the adult this innervation would be sparse. However, the immunocytochemical evidence presented is likely to have greatly underestimated both the spatial extent and bouton density of SPF cell axons (Figure 8A). In the earlier stages of PFC maturation such connectivity is probably more abundant and widespread with a possible convergence of SPF cell output into specific (mini) columns — perhaps onto select populations of pyramidal cells (see below).

Furthermore, the small proportion of non-spiny (smooth) dendritic shafts innervated by SPF cells (Table 1) likely belong to CRet-/GABA+ layer 1 LCNs (Figures 1D,F, 14H). In rat, these GABAergic neurons have been shown to furnish not only extensive lateral axonal arbors in layer 1 but also vertical axons innervating superficial pyramidal cells and LCNs, as well as pyramids deeper in the cortex (Muralidhar et al., 2014; Lee et al., 2015).

### Persisting SPF cells as “Integrative Hubs”

The presence of SPF cells with normal structural integrity in young adult and adult monkey PFC suggests that these neurons remain functional (Figure 11). SPF cells are immersed in the same extrinsic and intrinsic afferent pathways as their main synaptic targets — the feltwork of apical dendritic tufts in layer 1 (Figure 15B). However, SPF cells, with their polarized fan-like dendritic arbors, may be privy to specific combinations and strengths of inputs (Figures 15A,C). Of functional significance is the fan-like architecture of SPF cell dendrites and their short dendritic thorns. The latter structures receive numerous A-type and S-type inputs (Figure 12A) and have the electromorphometry to significantly influence processing mechanisms in parent dendritic shafts (Han and Heinemann, 2013; Chabrol et al., 2015). Therefore, SPF cells (together with neurons of the Cajal-Retzius family and other excitatory neurons in layer 1) could act as local internuncial hubs responsible for feed-forward excitation affecting the integration of neural information within the apical dendritic tufts of target pyramidal cells (Figures 15B,C). These apical tufts would be derived from many rows of minicolumns (Figure 15B). This strategic role would influence intra-/interareal and subcortical pyramid projection pathways from discrete patches of cortex (Figure 15B). It is proposed that such regions could be finalizing the developmental refinement of synaptic circuitry subserving emotional and cognitive processes during the protracted post-natal maturation of primate PFC (Petanjek et al., 2011; Bianchi et al., 2013; Amlien et al., 2016).

In conclusion, this study identified eight classes of CRet+ neurons (including Cajal-Retzius cells) in layer 1 of adult macaque monkey PFC, with a morphologically distinct type of CRet+/GABA- neuron, named “subpial fan (SPF) cell,” described comprehensively. SPF cells may be related to the persisting cells identified by Ramón y Cajal and by Retzius in humans. A full characterization of this rare neuron class, and its potential role in the developmental trajectory of primate cortex is open to future combined genetic, molecular, anatomical and electrophysiological research.

## Author contributions

The author designed and undertook the research, acquired and analysed the data, wrote the manuscript and approved it for publication.

## Funding

This work was supported by the Wellcome Trust (Grant: 047314/96/Z/WRE/MB), Royal Society (Grant: RSRG15788) and by The Research Committee of The Open University.

### Conflict of interest statement

The author declares that the research was conducted in the absence of any commercial or financial relationships that could be construed as a potential conflict of interest.

## Footnotes

^1^Ramón y Cajal (1899b) alludes to the “*Retzius'sche Zellen* of “Kölliker.” [See DeFelipe and Jones (1988) p.198].

^2^For reference: Translations of Ramón y Cajal's accounts of the “*Horizontal or special cells of the first layer”* and “*Cells of the plexiform layer with short axon”* in new born human motor cortex are provided by DeFelipe and Jones (1988, pp201–210).
